# A knowledge graph approach to predict and interpret disease-causing gene interactions

**DOI:** 10.1186/s12859-023-05451-5

**Published:** 2023-08-29

**Authors:** Alexandre Renaux, Chloé Terwagne, Michael Cochez, Ilaria Tiddi, Ann Nowé, Tom Lenaerts

**Affiliations:** 1 Interuniversity Institute of Bioinformatics in Brussels, Université Libre de Bruxelles - Vrije Universiteit Brussel, Brussels, Belgium; 2 Machine Learning Group, Université Libre de Bruxelles, Brussels, Belgium; 3 Artificial Intelligence lab, Vrije Universiteit Brussel, Brussels, Belgium; 4 Computer Science, Vrije Universiteit Amsterdam, Amsterdam, The Netherlands; 5 Discovery Lab, Elsevier, Amsterdam, The Netherlands

**Keywords:** Disease genetics, Genetic interactions, Interpretable machine-learning, Knowledge graphs

## Abstract

**Background:**

Understanding the impact of gene interactions on disease phenotypes is increasingly recognised as a crucial aspect of genetic disease research. This trend is reflected by the growing amount of clinical research on oligogenic diseases, where disease manifestations are influenced by combinations of variants on a few specific genes. Although statistical machine-learning methods have been developed to identify relevant genetic variant or gene combinations associated with oligogenic diseases, they rely on abstract features and black-box models, posing challenges to interpretability for medical experts and impeding their ability to comprehend and validate predictions. In this work, we present a novel, interpretable predictive approach based on a knowledge graph that not only provides accurate predictions of disease-causing gene interactions but also offers explanations for these results.

**Results:**

We introduce BOCK, a knowledge graph constructed to explore disease-causing genetic interactions, integrating curated information on oligogenic diseases from clinical cases with relevant biomedical networks and ontologies. Using this graph, we developed a novel predictive framework based on heterogenous paths connecting gene pairs. This method trains an interpretable decision set model that not only accurately predicts pathogenic gene interactions, but also unveils the patterns associated with these diseases. A unique aspect of our approach is its ability to offer, along with each positive prediction, explanations in the form of subgraphs, revealing the specific entities and relationships that led to each pathogenic prediction.

**Conclusion:**

Our method, built with interpretability in mind, leverages heterogenous path information in knowledge graphs to predict pathogenic gene interactions and generate meaningful explanations. This not only broadens our understanding of the molecular mechanisms underlying oligogenic diseases, but also presents a novel application of knowledge graphs in creating more transparent and insightful predictors for genetic research.

**Supplementary Information:**

The online version contains supplementary material available at 10.1186/s12859-023-05451-5.

## Background

In recent years, the field of medical genetics has seen a shift away from the traditional Mendelian model of genetic inheritance, challenged by the emergence of human disorders that exhibit incomplete penetrance, high phenotypic variability or locus heterogeneity [1–5].

This has led to the consideration of alternative genetics models, one of them being the oligogenic model, where a combination of causative variants is distributed among two or a few genes [6–9]. This model represents a bridge between the traditionally considered monogenic and the poorly understood polygenic or complex disorders [10–12].

An increasing number of clinical studies have reported evidence of oligogenic etiologies in various diseases, some of them previously considered as strictly monogenic. While some diseases may be strictly caused by the combined effect of multiple genes, others involve modifier genes that can affect the severity or presentation of the condition [13, 14]. The Oligogenic Disease Database (OLIDA) [15] provides a centralised resource for these studies. It collects genetic and clinical information from these reported cases and assigns curation scores based on multiple types of genetic and functional evidence. Meta-analyses from these studies revealed that genes associated with oligogenic diseases interact in intricate, epistatic ways [16] and exhibit a diverse range of functional relationships involving both direct and long-range interactions [6, 17, 18]. Nevertheless, the precise causal mechanisms behind most oligogenic diseases remain unresolved.

Previous work has established predictive methods for the pathogenicity of variant combinations [19], as well as the likelihood that certain gene pairs may produce a digenic disease [20]. These tools, while reporting a good predictive performance in cross-validation and independent validation tests, demonstrate nonetheless limited interpretability owing in part to the structural complexity of the models (e.g. a random forest consisting of many deep trees), and the abstract and continuous nature of features, most of them derived from complex bioinformatics methods (e.g. CADD score [21] or recessiveness probability [22]). While such models may further provide a ranking of the factors contributing the most to a given prediction, they provide little information about the molecular associations and functional patterns driving the disease, such as compensatory and synergistic mechanisms [23–26]. Additional methods are thus required to translate predictive elements into biologically meaningful information and relationships by leveraging prior knowledge, as demonstrated in [27]. This need aligns with recent efforts that have effectively harnessed background knowledge to uncover potential causal mechanisms behind molecular signatures derived from high-throughput experiments [28–30].

Biological and molecular prior knowledge is commonly represented as networks, capturing the physical and functional relationships between entities. Knowledge graphs (KGs) take this a step further by integrating diverse networks and ontologies into a single, comprehensive graph. KGs leverage semantically-rich relationships and contextual connections to generate valuable insights. Integrated networks within KGs have demonstrated superior performance in prioritising novel disease-gene associations [31–33] and are considered as a promising strategy to handle the data scarcity in rare diseases [34]. However, predictive methods on KGs often require transforming the original network into a homogeneous structure [35] or embedding it into a latent space [36], resulting in potential information loss and reduced interpretability [37].

Path-based approaches on KGs represent a promising avenue for inferring new relationships transparently, providing meaningful explanations for these predictions. For instance, the RPath method [29] reasons over paths within a knowledge graph, guided by transcriptomic information, to prioritise drugs for a given disease and reveal targeted proteins along these paths. In the context of machine learning, path information has also been employed for rule inference in KGs [38–40], enabling the prediction of new facts with a high degree of interpretability [41, 42]. In particular, these methods can capture path information at a more abstract level, by recording their sequence of node and edge types, also known as metapaths [43, 44]. Metapaths have been employed to generate features from KGs for various classification tasks. For example, metapath features derived from Hetionet, a large biomedical KG, have been applied in gene-disease prioritization [45] and drug repurposing [46].

In this work, we leverage a biological KG to i) mine association rules from frequently observed sets of metapaths in pathogenic gene pairs, ii) use these rules to identify novel pathogenic genetic interactions, while iii) providing a fully interpretable model and graphical explanations for each prediction.

To that end, we construct a new KG integrating known oligogenic disease information within relevant and trusted multi-level biological networks. Our new framework mine complex association rules based on metapaths found in disease-causing gene pairs. These rules are combined as a decision set model [47], which transparently predicts potential pathogenic gene interactions.

Our model can accurately identify gene interactions beyond known disease-related genes. Importantly, this method is interpretable, providing graph-based explanations from the KG. This form of explanation, grounded in biological knowledge, can foster trust, aid expert assessment [48], and can help in formulating new hypotheses for understanding gene interactions and disease mechanisms.

## Results

### BOCK: a knowledge graph integrating oligogenic diseases with biological networks

Using the oligogenic information from the clinical literature present in OLIDA and multiple public biological network resources, we constructed BOCK (Biological networks and Oligogenic Combinations as a Knowledge graph), a KG that puts oligogenic combinations into a biological context. Compared to more generic KGs, we selected specifically networks relevant to understanding the molecular mechanisms of epistasis, placing genes as the central entities, and focusing on trusted resources describing a large set of human genes and their interactions.

**Fig. 1:**
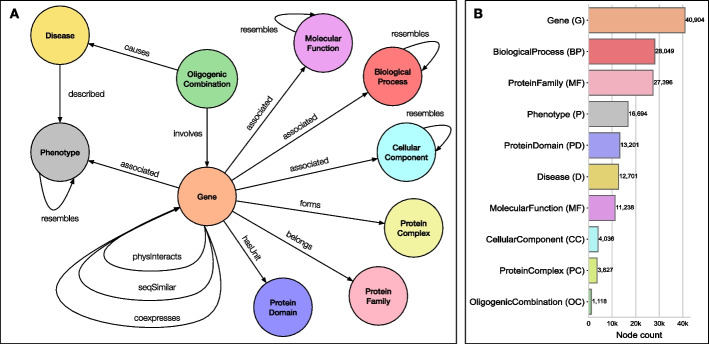
Schema and node statistics of the KG (BOCK). **A** KG schema representing the different node types (i.e. metanodes) as circles and their relationships as arrows (bidirectional arrows indicate undirected associations). **B** Number of nodes in the KG per metanode. We define an abbreviation for each metanode, in parenthesis, to simplify all metapath and rule notations in the following sections (see also Table 1)

This new resource comprises 158,964 nodes of 10 different types (Fig. 1B) and 2,659,064 edges of 17 different types (Table 1), structured according to the schema presented in Fig. 1A. We provide the complete KG open-access, in semantically rich formats that facilitate its exchange and use (see Availability of data and materials section).

**Table 1:** Knowledge graph edge types

Metaedge	Abbreviation	$\#$ Edges	$\#$ Sources	$\#$ Targets
Gene–coexpresses–Gene	GeG	1,338,764	14,940	14,940
Gene–physinteracts–Gene	GpG	329,801	17,062	17,062
Disease–described$\rightarrow$Phenotype	DdP	233,175	12,676	10,423
Gene–associated$\rightarrow$Phenotype	GaP	209,416	4870	9151
Gene–seqsimilar–Gene	GsG	186,445	12,226	12,226
Gene–associated$\rightarrow$BiologicalProcess	GaBP	93,676	16,323	10,570
Gene–associated$\rightarrow$CellularComponent	GaCC	58,432	16,978	691
Gene–belongs$\rightarrow$ProteinFamily	GbPF	45,454	19,657	11,187
Gene–associated$\rightarrow$MolecularFunction	GaMF	43,331	14,540	4042
Gene–hasunit$\rightarrow$ProteinDomain	GuPD	41,314	15,828	6636
BiologicalProcess–resembles–BiologicalProcess	BPrBP	33,102	10,811	10,811
Phenotype–resembles–Phenotype	PrP	16,000	7681	7681
Gene–forms$\rightarrow$ProteinComplex	GfPC	14,531	4357	3604
MolecularFunction–resembles–MolecularFunction	MFrMF	11,239	3710	3710
OligogenicCombination–involves$\rightarrow$Gene	OCiG	2700	1118	907
OligogenicCombination–causes$\rightarrow$Disease	OCcD	1173	1118	175
CellularComponent–resembles–CellularComponent	CCrCC	793	483	483

Each type of edge (i.e. metaedge) in the KG is defined uniquely by its source and target node types with the relationship name in between. Directed metaedges are indicated by an arrow on the relationship. We define abbreviations for each metaedge to simplify further notations. The table presents statistics on the number of corresponding edges, source nodes and target nodes for each metaedge, ordered by decreasing number of edges

The corresponding source databases for these networks were selected based on their quality, accessibility, and interoperability. Some edge types were filtered before integration, allowing only the connections with a minimal level of confidence provided in the original network resource. Considering that studies on oligogenic cases rarely report the specific impacted proteins and to simplify the model, BOCK collapses genes and their associated proteins as a single entity type “Gene” (see BOCK data integration and resources in Methods). The non-redundant contribution of each network source for genes is detailed in Additional file 1: Table A3.

The integration of OLIDA information together with the multi-scale biological networks enables the discovery of complex patterns that could not be found when considering each individual network in isolation. In this work, we focus on patterns mined from both direct and long-range relationships between pairs of genes associated with oligogenic diseases.

**Fig. 2:**
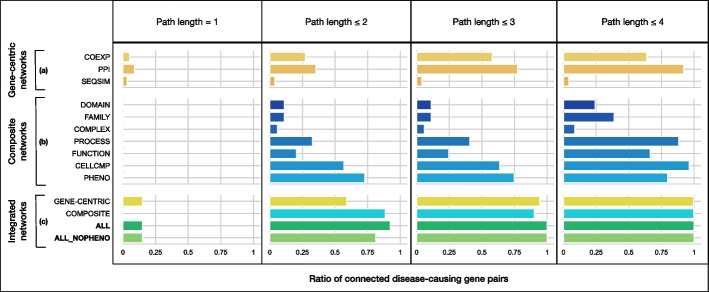
Ratio of connected disease-causing gene pairs in different components of BOCK. A gene pair is considered connected if there exists a path between the two genes, regardless of directionality, that can be traversed given a path length cutoff. Nodes of types “Disease” and “OligogenicCombination” were excluded and BOCK was decomposed into: (a) gene-centric networks (COEXP: Gene-coexpresses, PPI: Gene-physInteracts, SEQSIM: Gene-seqSimilar) shown merged as GENE-CENTRIC; (b) composite networks (DOMAIN: Gene-hasUnit-ProteinDomain, FAMILY: Gene-belongs-ProteinFamily, COMPLEX: Gene-forms-ProteinComplex, PROCESS: Gene-associated-BiologicalProcess, FUNCTION: Gene-associated-MolecularFunction, CELLCMP: Gene-associated-CellularComponent, PHENO: Gene-associated-Phenotype) shown merged as COMPOSITE; (c) integrated networks (GENE-CENTRIC: merge of (a), COMPOSITE: merge of (b), ALL: merge of (a) and (b), ALL$\_$NOPHENO: a subset of ALL excluding paths traversing “Phenotype” nodes). The ratios of connected oligogenic gene pairs with at least a weak evidence-level (Additional file 1: Table B2) are presented for these components according to path length cut-offs ranging from 1 to 4

As visualised in Fig. 2a, b, confident disease-causing gene pairs (i.e. those with at least a weak evidence level) exhibit only a partial connectivity (i.e. presence of at least one path between genes of a pair) when considering each BOCK component individually (see component details in Additional file 1: Table A2), indicating that single network approaches would be insufficient to support all cases.

Fusing these KG components as single graphs (Fig. 2c, GENE-CENTRIC; COMPOSITE) enables the connection of a majority of oligogenic gene pairs, when considering 3 and 4-hop paths. When integrating both gene-centric and composite graphs (ALL in Fig. 2), paths of lengths $\le$ 3 are sufficient to connect all oligogenic gene pairs. This observation remains true when excluding the Phenotype information (ALL_NOPHENO), prone to study biases for oligogenic gene pairs, as discussed in the following results.

### A framework to discover predictive rules from knowledge graph paths

**Fig. 3:**
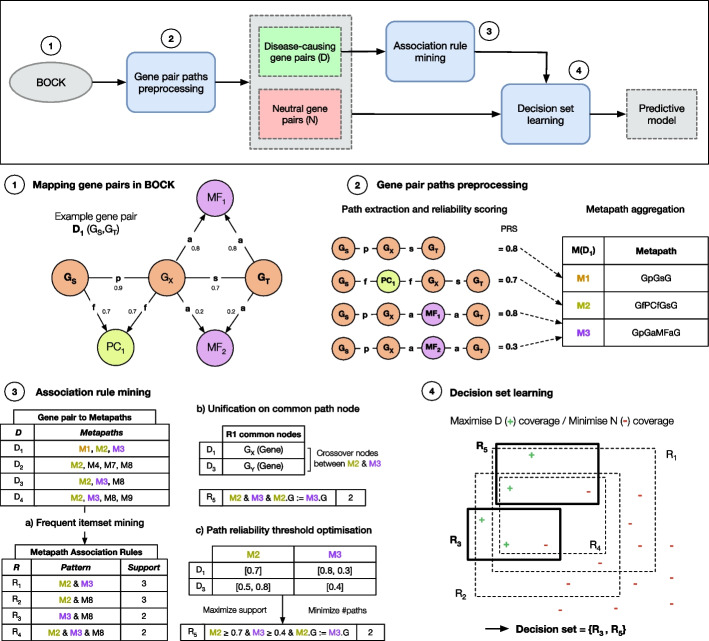
KG-based associative classifier training workflow. The diagram outlines how our framework uses labelled gene pairs and the path information in BOCK to train a rule-based model predicting pathogenic gene interactions. **(1)** Given a disease-associated gene pair (represented as $D_1$ ($G_S$,$G_T$)), all paths in BOCK starting at the gene node $G_S$ and ending at the gene node $G_T$ are collected, up to a certain predetermined path length cutoff. Although this traversal disregard edge directionality, the original direction of the edges is encoded in the recorded paths; **(2)** Each path is attributed a reliability score based on the original edge weight. Paths are then aggregated into their metapaths (i.e. path types) (M); **(3.a)** Association rules (R) are mined by finding frequent patterns of metapaths occurring in disease-causing gene pairs (D). Rules are extended with additional metapath conditions as long as their support (i.e. the weighted frequency of the pattern) is greater than a defined threshold (*minsup*); **(3.b)** Rules can be extended with a unification condition (e.g. node $G_X$ common to metapaths *M*1 and *M*2) if such pattern remains frequent; **(3.c)** The mined rules are refined with path reliability thresholds aiming to filter paths of lower quality while preserving a high rule support; **(4)** Using all pre-mined rules (R) and training data made of disease-causing gene pairs (D) as positive examples and a set of putative neutral gene pairs (N) as negative examples, a decision set model is trained by selecting a subset of predictive rules

Building upon our knowledge graph BOCK, we extended its capabilities with the creation of ARBOCK (Association Rule learning Based on Overlapping Connections in Knowledge graphs). This innovative approach specifically harnesses the characteristics of paths linking potential pathogenic gene pairs within the knowledge graph to construct a rule-based classification model (Fig. 3). Built on associative classifier principles [49–51], this two-step model starts by generating a rule set from local patterns of the pathogenic gene pairs (Fig. 3(2), (3)). The second step combines these rules into a decision set (DS) classifier [47], enabling identification of potential pathogenic gene pairs (Fig. 3(4)). This approach was chosen to balance interpretability and performance.

The model’s parameters, summarised in Additional file 1: Table C1, can be adjusted to manage computational complexity and the volume of discovered patterns. These parameters were empirically determined in this study to optimise predictive performance while minimising explanation complexity (Additional file 1: Figures in Appendix C).

Our approach starts by traversing all paths in BOCK, that connect a specified starting gene to a targeted ending gene, up to a predetermined path length cutoff (path_cutoff). For this study, we set this length cutoff to 3, which ensures full connectivity among known pathogenic gene pairs (Fig. 2). Although the traversal does not consider the original edge directions, the original edge directionality is retained within their encodings. For this study, genes are ordered according to the Residual Variant Intolerance Score (RVIS) and paths traversing “OligogenicCombination” and “Disease” nodes are ignored, as these inherently contain the answer to our predictive problem. For each path, a path reliability score is attributed based on the geometric mean of its edge scores and paths are then grouped into corresponding path types, or metapaths (Fig. 3(2); see Path traversal and confidence scoring in Methods).

Next, association rules are mined from the positive set only using the Apriori algorithm [52]. This algorithm finds sets of metapaths that frequently occur together (Fig. 3(3.a)). A rule is deemed frequent if its support is greater than or equal to a predetermined threshold (minsup_ratio, set to 0.2 for this study). The number of metapaths in a rule is limited (max_rule_length, set to 3 in this study) and redundant rules are removed. Rules can be optionally extended with a unification condition, which involves a common node between paths of at least two metapaths (Fig. 3(3.b)) (see Association rule mining on paths in Methods). Subsequently, rules are optimised with path reliability thresholds to improve interpretability and limit potentially spurious paths (Fig. 3(3.c); see Optimisation of path reliability thresholds in Methods).

The final stage of our approach involves training a decision set (DS) classifier [47] (Fig. 3(4)). This algorithm aims to find a subset of rules maximising the coverage of positive instances and minimising the coverage of negative instances (which balance is determined by the $\alpha$ parameter, set to 0.5 for this study). The trained DS model takes a gene pair and its associated BOCK-paths as input and returns a probability of pathogenicity along with the matched rules, if any (see Training of a decision set classifier in Methods).

We implemented this framework as a Python package and made available all scripts to replicate the subsequent results. Users can evaluate selected gene pairs on pre-trained models and train new models following the same methodology (refer to the Availability of data and materials section). The current implementation, tested on an Intel Core i7, is capable of retrieving path data from BOCK at an average speed of 1.16 s (std. 2.2) for each gene pair (using path_cutoff=3), and produces predictions along with corresponding explanations at an average rate of 0.5 milliseconds (std. 2.9) for each gene pair. Excluding the path retrieval, the training of a new model (rule mining + decision set learning), using the parameters and the full data presented in this study, took an average of 29.26 mn parallelised on 8 cores.

In this study, we applied our methodology on two labelled training datasets: a positive set (D) of 441 disease-causing gene pairs with established familial or statistical evidence of pathogenicity, and a negative set (N) of 44,100 putative neutral gene pairs, selected from a cohort of healthy individuals. Each gene pair is given a weight signifying the confidence in its label. Detailed process for these sets’ creation is described in the Gene pair selection criteria Methods section.

We also assessed two distinct DS models. The first model considers all valid paths (designated as *incl.Pheno*), whereas the second excludes paths involving Phenotypes (*excl.Pheno*). The practical implementation of this methodology and its effectiveness for this application are demonstrated in the subsequent sections.

### Rule mining detects prevalent patterns in oligogenic combinations

Considering only the rule mining part of our framework and the previously defined parameters (Additional file 1: Table C1), we now explore common patterns occurring in the 426 pathogenic gene pairs from the training dataset. These patterns, represented as rules, are integral to the following predictive modelling stage. Each rule is formulated as a set of conditions, involving multiple metapaths, associated to a class label (here, the disease-causing label $l_D$). The predictive power of each rule is estimated through its confidence metric – the likelihood of a gene-pair being pathogenic when it satisfies the conditional clause – assessed against both the disease-causing (*D*) and neutral (*N*) sets.

**Fig. 4:**
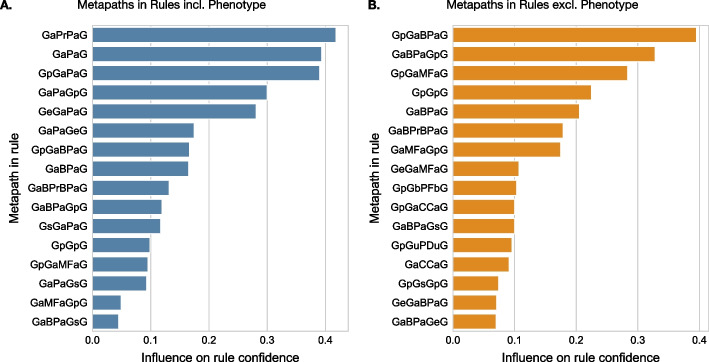
Metapath influence on rule confidence Metapaths significantly associated with higher rule confidence are shown. Rule confidence distributions are compared for each metapath, considering both its presence and absence as a rule condition. The significance of the difference is determined using a one-tailed Wilcoxon ranksum test with Bonferroni correction (adjusted p-value $\le 0.01$). Metapaths are ranked based on their effect size, measured by the rank-biserial correlation. **A** Rules mined from paths including Phenotype (*incl. Pheno*). **B** Rules mined from paths excluding Phenotype (*excl. Pheno*)

To better understand the relationship between the rule metapath content and its predictive power, we analysed metapaths significantly associated with higher rule confidence, enabling us to shed light on the most influential types of relationships (Fig. 4). The confidence distribution of the rules, in relation to their support, can be found in Additional file 1: Fig. D1.

Upon considering all valid paths (*incl. Pheno*), 6917 rules were mined. Analysing the rules’ conditions, 16 metapaths emerged as significantly associated with higher confidence rules (Fig. 4A). High-confidence rules often include metapaths related to similar phenotypes ($50\%$), biological processes ($31.2\%$), and molecular functions ($12.5\%$), and typically involve intermediate genes ($75\%$) linked with a diverse range of relationships. Metapaths containing phenotype information, specifically *GaPaG* and *GaPrPaG* (reflecting common and related phenotypes between gene pairs), hold the most influence among high-confidence rules.

Conversely, excluding paths traversing Phenotype nodes (*excl. Pheno*) resulted in 4124 rules. Analysis of the rules’ conditions revealed 16 metapaths significantly associated with higher confidence rules, of which 8 were unique to this setting (Fig. 4B). High-confidence rules predominantly present heterogenous metapaths associated with a wider range of entities including biological processes ($43.8\%$), molecular functions ($18.8\%$), cellular components ($12.5\%$), protein families ($6.2\%$), and protein domains ($6.2\%$). Metapaths related to biological processes and molecular functions are most influential. Particularly, *GpGaBPaG* and its reverse, which capture shared processes between a gene pair and an interacting gene, stand out in the highest confidence rules.

### An optimal set of rules can identify potential pathogenic gene pairs

Using our new approach with empirically determined parameters (see full analysis in Appendix C and the summary of selected parameters in C1), we trained decision set (DS) models based on 426 pathogenic gene-pairs and 42,600 neutral gene pairs (imbalance ratio at 1:100), setting aside 15 recently published and high-quality pathogenic gene pairs for independent testing. (see Gene pair selection criteria in Methods).

We analysed two DS models: *DS incl.Pheno* (including Phenotype-traversing paths) and *DS excl. Pheno* (excluding Phenotype-traversing paths). Both were evaluated under a stratified 10-fold cross-validation and tested on the independent test set. We reported essential information to assess this machine learning approach following the DOME recommendations [53] in Additional file 1: Table E1.

**Fig. 5:**
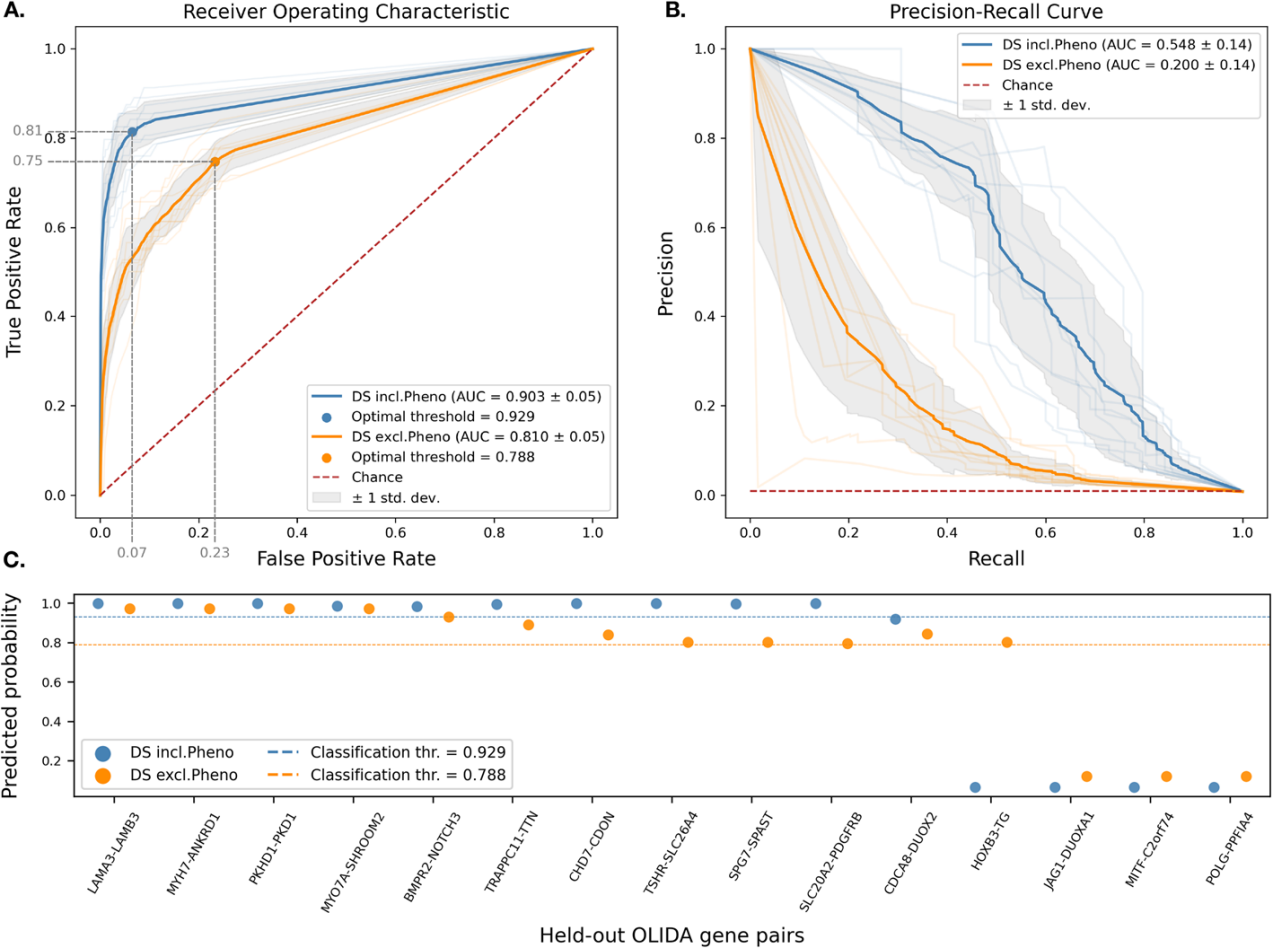
Decision set models performance. Two DS models have been evaluated: one trained with all valid paths (*DS incl.Pheno*) and one trained without Phenotype-traversing paths (*DS excl. Pheno*), on a stratified 10-fold cross-validation setting. A test set of 15 pathogenic gene pairs from recent literature has been held out for independent evaluation. **A** Receiver operating characteristic (ROC) curve obtained by averaging all fold curves. The best classification threshold is evaluated using the geometric mean between the sensitivity and the specificity. **B** Precision-Recall curve obtained by averaging all fold curves. **C** Test set predicted probabilities, displayed in decreasing order. The horizontal lines represent the optimal thresholds for binary classification

The performance of these models is depicted in Fig. 5. The *DS incl.Pheno* model achieves an AUROC of 0.903 (std. 0.03) and AUPRC of 0.548 (std. 0.07), recalling $81.8\%$ of pathogenic gene pairs with a $6.6\%$ false positive rate at the optimal threshold. The final model, consisting of 35 rules, successfully predicts 10/15 held-out pathogenic gene pairs. In comparison, the *DS excl.Pheno* model achieves an AUROC of 0.810 (std. 0.03) and an AUPRC of 0.200 (std. 0.07), recalling $75.9\%$ of pathogenic gene pairs with a $24.1\%$ false positive rate at the optimal threshold. The fully trained model, consisting of 27 rules, identifies 12/15 held-out pathogenic gene pairs.

For baseline comparison, we compared these to a simpler model based on the random walk with restart (RWR) probability from the knowledge graph (restart probability = 0.7) [37]. Our analysis, detailed in Additional file 1: Fig. F1, demonstrates that while the graph’s topology alone can predict disease-association patterns, our approach, by harnessing the semantics of heterogeneous paths, outperforms this baseline model (*incl. Pheno*: mean AUPRC=0.159, *excl.Pheno*: mean AUPRC=0.084).

While the model incorporating phenotype information delivers promising results, its limitations must be considered. Specifically, the disproportionate Gene-Phenotype annotation coverage in disease-associated genes could introduce bias in machine-learning models trained on this dataset. While only $23.5\%$ of all human genes are linked to a phenotype term, the proportion rises notably to $82.6\%$ when considering only genes involved in known oligogenic diseases (see Additional file 1: Fig. G1).

This suggests that models trained with phenotype association features could primarily make decisions based on these features and thus, disproportionately identifying gene pairs from the limited $23.5\%$ pool of phenotype-annotated genes. To illustrate the effect of this bias, we examined the Digenic Gene Predictor (DiGePred) [20], a statistical machine-learning method that reports high-accuracy (average AUROC of 0.972 in cross-validation) in predicting pathogenic gene pairs. This model assigns 44% of feature importance to phenotype-based characteristics, indicating a strong reliance on such features. We evaluated this model on our independent test set comprised of recently published instances (*i.e* potentially less affected by such knowledge bias). DiGePred was able to correctly identify 4/15 gene pairs, all of which were fully annotated with phenotype terms. The remaining gene pairs often lacked complete phenotype annotation (6/11) or had limited common phenotype terms (Jaccard Index between 0.01 and 0.11), making them harder to identify by this model (see Additional file 1: Table G3).

Similarly, the phenotype-inclusive model (*DS incl. Phenotype*) exhibits a similar bias (Additional file 1: Fig. G2). However, this model can nonetheless capture indirect Phenotype relationships due to the metapath-based design of its rules. This property enables the coverage of a wider pool of genes than methods relying on direct Phenotypic associations only (see example of the gene pair *MYO7A-SHROOM2* in Additional file 1: Table G3).

We extended our analysis to evaluate the impact of removing various types of relationships (i.e., metaedges) in BOCK beyond just Phenotype relationships. Detailed results, provided in Additional file 1: Appendix H, show that eliminating physical interaction edges (*GpG*) and associations with biological processes (*GaBP*) adversely affects classifier performance. Removing other metaedges has a negligible effect on predictive performance. Interestingly, omitting gene functional relationships results in more intricate graphical explanations (see next section), possibly due to an increased prevalence of gene-gene relationships in rules. The removal of the coexpression relationship *GeG* reduces the number of paths in the provided explanations, likely owing to the high frequency of *GeG* edges in BOCK (see Table 1).

### Metapath-based rules highlight relevant paths as predictive explanations

The predictive model presented in this work offers both global interpretability and context-specific explanations for pathogenic gene pairs. On the one hand, the simplicity of this model model allows users to examine all the rules that contribute to a pathogenic prediction. On the second hand, it provides transparent predictions by returning the matching rules associated with each predicted pathogenic gene pair.

Most importantly, all the rules from our model can be translated into a KG query, which retrieves a manageable subset of paths from BOCK. This subgraph contains concrete relationships and entities that can assist in generating hypotheses about the potential molecular mechanisms underlying the disease of interest.

**Fig. 6:**
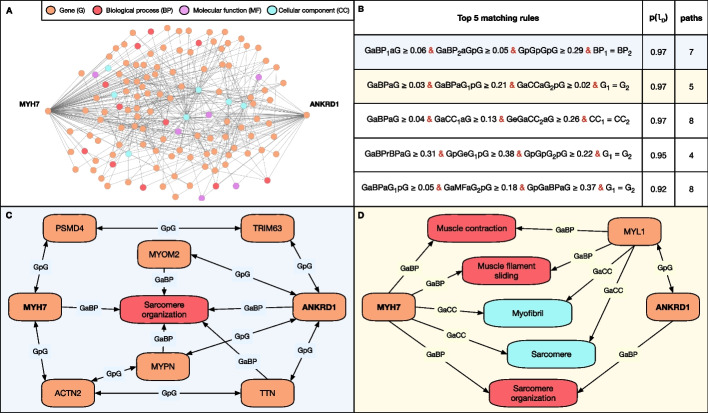
Predictive explanations generated by querying matching rules on the KG This figure showcases the example of the digenic gene pair *MYH7*-*ANKRD1*, part of the independent test set and predicted as disease-causing with the highest probability. **A** Subgraph extracted by traversing all paths (excluding those traversing “Phenotype”, “Disease” and “OligogenicCombination” nodes) of a length $\le$ 3. A total of 342 paths, 127 nodes and 447 edges exists. **B** Top 5 matching rules ranked by their associated probability score. Each rule is written in their abbreviated form (see Table 1) with its conditions separated by &. Indices for node types (e.g. BP$_1$) are used in unification conditions (e.g. BP$_1$=BP$_2$) to constrain entities to be the same across different metapaths. The numerical value associated with each metapath (e.g. $\ge$ 0.21) sets the path reliability threshold, which conditions the minimum path reliability score of all underlying paths. We display the number of paths obtained by querying the KG with the rule with that specific gene pair. **C** Returned explanation subgraph for the 1st rule based on the 7 matching paths. **D** Returned explanation subgraph for the 2nd rule based on the 5 matching paths. Entity types are represented with the same colors as in (**A**). Explanations subgraphs for the 3rd, 4th and 5th rules are provided in Additional file 1: Figs. I

As an example, we consider the gene pair *MYH7*-*ANKRD1* from the independent test set, which was predicted as pathogenic with a high probability by both decision set models. Previous studies have demonstrated the involvement of this gene pair, with a digenic pattern, in Left ventricular noncompaction disease (LVNC) (ORPHA:54260; HP:0030682) associated with Dilated cardiomyopathy (DCM) phenotype (HP:0001644) based on familial evidence [54]. Exploring paths of up to length 3 between these two genes in BOCK (excluding “Phenotype,” “Disease,” and “OligogenicCombination” entities) reveals a large subgraph comprising 342 paths, 127 nodes, and 447 edges (Fig. 6A).

The DS *excl.Pheno* model applied to the *MYH7*-*ANKRD1* gene pair returns matching rules ranked by their associated probabilities. Figure 6B displays the top 5 rules along with the number of paths retrieved by querying the KG. Due to stringent path thresholds and the use of unification conditions, each of the top rules yields only a few paths. The first two rules are showcased in Fig. 6C, D, providing graphical explanations.

The first rule corresponds to a pattern where both genes of the combination share a common biological process (GaBP$_1$aG) and where a third gene, physically interacting, is also involved in the same biological process (GaBP$_2$aGpG; BP$_1$=BP$_2$). Both genes from the pair are also linked with a long-range physical interaction.

The second rule describes a pattern where a central gene (G$_1$=G$_2$) physically interacts with the second gene while sharing a biological process (GaBPaGpG) and a common cellular component (GaCCaGpG) with the first gene. Both genes of the pair also share a common biological process (GaBPaG).

In 4 out of the 5 presented rules, functional entities associated with the sarcomere (GO:0030017, GO:0045214) are shown relevant both via direct and indirect paths (Fig. 6 and Additional file 1: Fig. I). Mutations in sarcomere protein genes have been linked to both LVNC and DCM diseases [55, 56]. Among traversed genes, *ACTN2* has been previously associated with LVNC [57], *TTN* to LVNC and DCM [58, 59] and *MYPN* to DCM [60]. The association of other genes postulates novel hypotheses for further exploration. For example, *MYL1*, *MYOM2*, *TRIM63* and *PSMD4* have been broadly associated with myopathies [61–64] but not directly to LVNC or DCM yet, and could therefore be considered as potential targets to investigate.

## Discussion

In this study, we introduced BOCK, a knowledge graph (KG) developed to contextualise oligogenic interactions within broader biological networks. This new resource, in combination with our associative classification framework, allows for the extraction of predictive rules based on path information between known pathogenic gene pairs in the KG. Notably, this approach predicts the pathogenicity of gene pairs with accuracy and provides relevant path-based explanations.

Previous methods often examined biological networks in isolation, using simplified quantitative measures such as graph distances or Jaccard indexes as features. By contrast, our unified KG-based strategy consolidates disparate resources and uncovers a wider range of semantically rich interactions. While individual biological networks can be noisy and incomplete, combining them into a KG mitigates these issues, enhancing network connectivity and substantiating the relationship evidence between gene pairs. Additionally, we focused on trusted high-quality sources to build BOCK and refined our rules with operators able to filter lower quality or uninformative paths.

The rules underpinning our model are generated using an association rule mining approach, with a focus solely on known oligogenic gene pairs and their frequency of occurrence. This unsupervised methodology allows exploration of larger feature space compared to traditional greedy algorithms, particularly beneficial for discovering interesting rules within imbalanced datasets [65]. The minimum support criterion prevents overfitting by excluding rare and non-generalisable patterns, ensuring that each rule is based on multiple instances of known pathogenic combinations. Additionally, the incorporation of unifications and path reliability thresholds further enhances the specificity and interpretability of the extracted patterns. However, to manage the extensive number of rules potentially generated, we imposed stringent constraints on the search space. Enhancements in computational efficiency or KG filtering approaches [66] could be considered to explore patterns beyond these limitations.

We chose to construct an associative classifier, specifically a decision set [47], known for their interpretability within machine learning models. These models have been shown to enhance classification accuracy compared to other rule-based models by uncovering global patterns during the mining stage [50]. However, it is important to acknowledge that our gene pair pathogenicity predictor, when excluding phenotype information, exhibits a relatively high false positive rate. This outcome may be due in part to the uncertainty surrounding our selection of neutral gene pairs, which relied on the frequency of potentially deleterious variant pairs in healthy controls. To address this issue, future work might involve more nuanced strategies for neutral pair selection, as well as the integration of gene or variant-level features, KG latent representations [67], or a combination of our approach with existing black-box predictors [68].

To mitigate potential bias stemming from the limited phenotype annotation coverage across human genes, we intentionally developed a model that excludes phenotype-related paths. Our method demonstrates the capability to handle indirect relationships, allowing the inclusion of a wider pool of genes beyond those directly linked by phenotypic associations. However, incorporating phenotype-based rules may favour well-studied gene pairs and overlook those located in less-explored regions of the phenotype annotation network. In light of these considerations, when exploring the oligogenic origins of diseases, employing both phenotype-inclusive and exclusive predictors could be advantageous to ensure a comprehensive analysis.

Our proposed approach stands out by providing contextual explanations based on a knowledge graph (KG), offering insights that are more meaningful and trustworthy compared to explanations based solely on abstract features [48]. By translating rules into KG queries, we transform the explanations from an abstract feature space into concrete entities and relationships with traceable provenance, enhancing interpretability for the end-user. However, applying this method in real-world cases comes with challenges, such as potential explanation complexity when querying dense regions of the KG and the presence of overlapping rules for some predictions. We also currently do not offer ways to validate explanations based on external sources of information. Further research should focus on developing systematic assessment methods to enhance the quality of explanations and address these challenges.

## Conclusion

In conclusion, our study highlights the potential of leveraging heterogeneous paths in knowledge graphs (KGs) for accurate and interpretable predictions of pathogenic gene interactions. Our novel predictive framework distinguishes itself from existing approaches by featuring: (1) a KG that serves as a hub for deriving all metapath-based features, (2) a rule-based model that transparently explains its decisions, and (3) context-based explanations that present relevant subgraphs tied to positive predictions. Although our present work primarily focuses on interaction patterns and interpretability, future research could further develop this model by including additional features or working in concert with statistical learning methods. Such advancements, when linked with explanation-focused approaches using knowledge graphs, could offer a deeper understanding of oligogenic diseases and provide clearer and more insightful genetic interaction predictions.

## Methods

### BOCK data integration and resources

We integrated multiple biological network and ontology resources, together with the information on known oligogenic diseases, into a KG called BOCK (Biological networks and Oligogenic Combinations as a KG). In KGs [69, 70], nodes and edges can be qualified with types, facilitating the integration of heterogeneous concepts into a single graph.

In our KG, nodes represent biological entities and biomedical concepts defined by a specific node type, a unique Uniform Resource Identifier (URI) linking the node to its source database entry, as well as optional node properties. Edges represent relationships between these entities, defined by a specific type and an optional confidence score, indicative of the quality or the strength of the relationship.

Sources for creating the KG were selected based on domain knowledge and according to multiple criteria: (1) Relevance in human disease aetiology: by considering multiple biological levels of organisation often affected in pathologies and by selecting strictly for human-derived data; (2) Quality control: by favouring resources based on clear curation policies and substantial accuracy in the case of electronically inferred annotations; (3) Gene coverage: by only considering resources linking at least 20% of all human genes; (4) Accessibility and interoperability: by choosing resources from public and free-to-use databases, attributing each entity with a unique and retrievable identifier. The KG integration is summarised in Additional file 1: Table A1, describing all source databases and their versions, and in Additional file 1: Table A2 describing how the source information is structured and pre-processed. The final schema of the KG is provided in the results, Fig. 1.

#### Oligogenic combinations

OLIDA aggregates curated information about oligogenic diseases gathered from the medical literature [15]. Each entry consists of a genetic variant combination involving several genes linked with contextual information, such as the associated disease, the source scientific article, the suspected oligogenic effect and its curation confidence scores.

The BOCK KG encodes the relational information from OLIDA by linking the involved “Gene” and “Disease” entities via a dedicated “OligogenicCombination” node pointing to the OLIDA identifier of a given oligogenic variant combination. Additional properties have been added to this node, such as the OLIDA curation confidence scores, the publication DOI and timestamp, the ethnicity of the associated patient and the suspected oligogenic effect [71].

#### Gene mappings

Considering that clinical studies on oligogenic cases rarely report the effect of variants on specific encoded proteins, we chose to reduce the model complexity of BOCK by collapsing all protein identifiers at the gene level into entities of type “Gene”. Gene and protein identifiers from all integrated resources were collapsed and mapped into their corresponding Ensembl identifiers. The databases Ensembl [72], UniProt [73] and HGNC [74] were used as a reference to handle potential identifier mapping ambiguities.

Edges linking protein pairs were also collapsed as edges between their associated genes, with an associated score computed as the maximum of all original scores.

Two properties were also added to the “Gene” entity: the Residual Variation Intolerance Score (RVIS) [75] and the human Gene Damage Index (GDI) [76], obtained from dbNSFP [77].

#### Protein interactions

Direct protein-protein interactions (PPI) were sourced from Mentha [78], a resource aggregating exclusively manually curated protein-protein interaction databases that have adhered to the IMEx consortium, with a particular emphasis on experimentally verified interactions compared to other PPI sources. The Mentha human interactome data was integrated into BOCK, establishing *physInteracts* edges between *Gene* entities, weighted by the provided Mentha reliability score.

#### Protein sequence similarity

Sequence similarity links were built using BLAST pairwise protein alignment bit scores [79] obtained from STRING [80]. In line with human homology detection recommendations [81], only proteins with aligned regions covering at least 50% of the shorter protein were considered. The Blast Score Ratio (BSR) was computed [82], bounding all alignment scores in the interval [0, 1], and edges with BSR values of at least 0.2, determined based on functional similarity signal [83], were included as “seqSimilar” types, linking “Gene” entities and weighted based on BSR.

#### Tissue co-expression

We extracted tissue-specific co-expression data from the TCSBN database [84]. Compared to other databases, TCSBN leverages GTEx’s comprehensive RNA-seq data [85] and offers detailed tissue-specific co-expression statistics, greatly enhancing the resolution of downstream analyses. To enhance signal strength, we applied several filters: (1) Tissues with fewer than 70 samples were excluded per GTEx recommendations, and redundant subtypes were consolidated. (2) Co-expression relationships involving a gene with a z-score below -3 in any given tissue, as per the standardised GTEx median tissue gene expression levels, were discarded [86]. (3) Edges were kept only if they exhibited significant adjusted p-values (< 0.01) and strong correlation ($\rho$ $\ge$ 0.80), adjusted by tissue sample size with the Fisher transformation [87]. These were integrated into the KG as type “Gene”, linked by “coexpresses”, and scored by the maximum correlation value across tissues. The “in” edge property records the set of tissues.

#### Protein domain and families

We extracted protein domain and family information, as well as their annotations on human proteins, from the InterPro database [88]. Entries from InterPro were integrated as “ProteinDomain” and “ProteinFamily” node types and linked to nodes of type “Gene” via edges of types “hasUnit” and “belongs” respectively.

#### Protein complexes

Protein complexes were extracted from the CORUM database [89], a resource of manually annotated protein complexes from mammalian organisms. Compared to other resources, CORUM exclusively provides species-specific data from curated publications, without any inference between organisms. We selected complexes found in human and integrated each complex as a “ProteinComplex” node linked, via an edge “forms”, to its sub-units corresponding “Gene” entities.

#### Phenotype and disease information

Phenotypic information from the Human Phenotype Ontology (HPO) [90] was integrated, focusing on all non-obsolete terms under the category “Phenotypic abnormality” as “Phenotype” nodes. These nodes were connected with respective “Gene” entities using the provided phenotype-gene annotations, creating “associated” edges.

Disease information, sourced from medical literature and reference disease databases such as OMIM and Orphanet [90, 91], was coupled with phenotype associations. A “described” edge was created between “Disease” and “Phenotype” entities, scored according to the frequency of the phenotype when available.

#### Gene ontology annotations

The Gene Ontology (GO) knowledge base’s three sub-ontologies — “BiologicalProcess” (BP), “Molecular Function” (MF), and “Cellular Component” (CC) — were integrated into the KG, excluding obsolete and root terms [92]. Human gene associations from the Gene Ontology Annotation (GOA) file were included, specifically retaining positive associations identified with qualifiers “enables”, “involved_in”, “is_active_in”, and “located_in”. We discarded non-curated or not biologically supported associations (evidence code IEA and ND, respectively). The refined associations were linked to “Gene” entities and the corresponding GO entity with an “associated” edge type.

#### Gene functional annotation relationships

In the KG, many entities linked to “Gene” correspond to functional annotation terms, represented as nodes of type: “ProteinDomain”, “ProteinFamily”, “ProteinComplex”, “Phenotype”, “BiologicalProcess”, “MolecularFunction” and “CellularComponent”.

We assigned a score to the edges between one gene and an annotation term, estimating how informative these relationships are. This score was determined by looking at how frequently an annotation term occurs on human genes, with infrequent terms receiving higher scores and more common terms receiving lower scores.

More formally, we defined a metric, the functional information (FI), given an annotation term $t \in T$ of a specific entity type. This metric corresponds to the information content of the term *t*, scaled by the maximum information content and is therefore bound in the [0, 1] interval (Eq. (1)). The information content of a term *t* is derived from the ratio of the count of genes associated with that term, *g*(*t*), and its subterms $t_s$, to the count of genes associated with all terms of the same entity type, *g*(*T*).

1 $$\begin{aligned} \textrm{FI}(t)= \frac{- \log \left( |g(t~\cup ~(\bigcup _{t_s \sqsubseteq t} t_s))|~/~|g(T)|\right) }{\log \left( \left| g(T)\right| \right) } \end{aligned}$$

#### Semantic similarity relationships

The KG incorporates the terms from two comprehensive ontologies, GO and HPO, structured as hierarchies of terms interconnected by semantic associations in the form of a directed acyclic graph. We considered the term subclass hierarchies, obtained by extracting all “is a” relationships, to compute semantic similarity links between each pair of terms. The semantic similarity is a measure taking into account the distance between terms in a subclass hierarchy, with a higher value indicating terms with a similar meaning. More specifically, we computed the SimGIC semantic similarity, based on the information content of ancestor terms, which has been shown to perform best when assessing similar gene sequences [93]. We integrated these semantic relationships with an edge of type “resembles” whenever the semantic similarity between two terms is higher than 0.5.

### Gene pair selection criteria

#### Disease-causing gene combinations

Familial and statistical evidence scores from OLIDA were used to attribute a weak, moderate, and strong evidence level for each pathogenic variant combination. A weight was attributed to these instances according to the three defined levels of confidence (Additional file 1: Table B1).

All variant combinations involving two genes satisfying at least a weak evidence level were considered, amounting to a total of 794 variant combinations. These were subsequently aggregated at the gene pair level, weighted by the maximum confidence level. A total of 441 disease-causing gene pairs (*D*) were selected after aggregation (Additional file 1: Table B2).

To provide an independent testing of the predictive models, 15 pathogenic gene pairs were held-out. This test set was selected based on an automatic procedure designed to favour diverse, confident and recently published cases: first, all disease-causing gene pairs were ranked by their first associated article publication date, then for each gene pair from the most recent to the oldest, gene pairs were chosen if their evidence level was at least Moderate (Additional file 1: Table B1) and if none of their genes overlapped with the previously selected ones. Details about the selected gene pairs are provided in Additional file 1: Table B3.

#### Neutral gene combinations

A neutral gene pair dataset (*N*) was collected from healthy individuals by selecting gene pairs frequently mutated with variants statistically similar to those observed in oligogenic combinations. Variants coming from 2490 healthy individuals from the 1000 genome project (1KGP) [94] were first filtered based on the criterion of minimum allele frequency (MAF) $\le$ 0.03.

The variants were collapsed at the gene level for each control individual, by retaining the maximum CADD score [95] for all variants in each gene, resulting in a list of candidate genes and their associated score for each individual. Gene pairs were then generated and filtered, based on their frequency, to ensure that each selected gene pair occurred in a minimum of 50 healthy individuals.

Gene pairs were further selected if their maximum CADD score was higher than 3.57, corresponding to the first quartile (Q1) of the distribution of maximum CADD scores for all gene pairs occurring in OLIDA. In order to prevent sampling too many gene pairs from the same linkage disequilibrium block, we further constrained neutral gene pairs to be sampled from different chromosomes or to have their genomic coordinates at least 10 kb apart.

We finally calculated a gene pair score by averaging the minimum CADD scores between genes over all patients. This score was used to rank the selected gene pairs in decreasing order and the top 44.100 gene pairs were selected from this ranking, resulting in an imbalance ratio of 1:100 between the disease-causing gene pairs and the neutral gene pairs, respectively. This score was also used to assign a weight to each gene pair.

### Rule discovery using KG paths

#### Path traversal and confidence scoring

The relational information between all selected gene pairs was captured via a path traversal of the oligogenic KG, a path filtering procedure and a subsequent aggregation of paths into path types, also known as metapaths.

In the initial phase of the method, a traversal is conducted over all potential paths within the oligogenic KG between each identified gene pair. Each path begins from the gene with the lowest Residual Variant Intolerance Score (RVIS) [75] and ends at the gene with the highest score. The traversal process is not constrained by the original edge directionality; however, this directionality is encoded within the resulting metapath. For this study, the path_cutoff parameter, limiting the maximum number of edges in a path, was set to 3.

In order to maintain consistency between the properties of edges traversed, certain paths were automatically discarded. This consistency filtering was, in practice, only applied to paths crossing multiple “coexpresses” edges, where the tissues specified in the “in” property of these edges were not compatible with each other.

Finally, the paths were aggregated into metapaths, a sequence of node types and edge types that records the semantic pattern of the relationship. The original path information was recorded for later use in the mining stage.

To be able to compare and rank paths following the same metapath based on the informativeness and quality of relationships, a path reliability score was also attributed to each path by computing the geometric mean of all composing edge scores.

#### Association rule mining on paths

Frequent associations of metapaths were extracted from the disease-causing gene pairs using a level-wise search based on the Apriori algorithm [52], designed for the efficient exhaustive discovery of frequent patterns over transactional data. These patterns are considered as class association rules (CAR) [49] in the form: *conditional pattern* $\rightarrow$ *class label*, with *disease-causing* ($l_D$) as the only class label.

Each mined rule *r* is associated with a support value based on the covered disease-causing gene pairs ${\mathcal {C}}_{r}(D)$. We set the minsup_ratio parameter controlling the minimum relative support to consider a rule valid to 0.2 for this study. Both support and minsup_ratio were adjusted according to the weight associated with each gene pair in order to give more importance to instances with higher confidence. For this study, the max_rule_length parameter was set to 3, limiting the maximum number of metapath conditions in a rule.

We extended the mining of simple metapath associations by searching for unification constraints between metapaths. Unifications are variables expressed in multiple conditions that can be substituted with the same value. This concept has been adapted to metapaths by searching for nodes at the intersection of paths associated with at least two different metapaths. Unified patterns were limited to one unification constraint and these patterns have the same minimum support constraint.

Finally, to minimise the redundancy of mined patterns, we only selected *closed itemsets*, by discarding all patterns where at least one of its superset pattern has the same support count [96].

#### Optimisation of path reliability thresholds

In order to limit the number of paths that could contribute to noise and reduced interpretability, previously mined rules were refined with conditions aiming to filter out paths below a certain path reliability score threshold. We implemented this refinement stage by searching, for every metapath composing a rule, a minimum threshold in the interval [0, 1] conditioning the associated paths in the training set to be scored with a value higher or equal to that threshold.

Note that, if high threshold values for a rule are set, fewer paths may be yielded, but the rule support may be decreased or the rule may even be invalidated if the support doesn’t meet the minimum support constraint; therefore, this search was implemented to take this tradeoff into account.

A differential evolution algorithm [97, 98] was used for this search, considering the non-linear problem to be solved. This algorithm works by evolving and combining a population of solutions (here, the optimal threshold values for a rule), retaining the most fit candidate solutions at each generation.

We implemented the fitness of a rule, given a set of thresholds $\Theta$, as defined in Equation (3). This fitness function is influenced positively by the rule support (*i.e* the coverage of disease-causing instances ${\mathcal {C}}_{r}(D)$) and negatively by the number of paths returned on matching instances $P_{r}(d)$ (with $d \in D$) on average (Eq. (2)). A fitness of 0 was returned for thresholds where the rule support was lower than minsup_ratio, to enforce the minimum support constraint. Gene pair weights were used to adjust both the rule support and the average number of paths calculation.

2 $$\begin{aligned} {\bar{\text{P}}}_r(D)&={} \sum \limits _{d\;\in \;D}\left| P_{r}(d)\right| /\left| {\mathcal {C}}_{r}(D)\right| \end{aligned}$$

3 $$\begin{aligned} \textrm{f}\left( r,\Theta \right)&={}\frac{1}{2}\cdot \left( \frac{\left| {\mathcal {C}}_{r|\Theta }(D)\right| }{\left| {\mathcal {C}}_{r}(D)\right| } + \left( 1 - \frac{{\bar{P}}_{r|\Theta }(D)}{{\bar{P}}_r(D)}\right) \right) \end{aligned}$$

This optimisation was performed with the differential evolution DE/best/1/bin scheme [99] shown to be the most accurate and robust strategy, regardless of the characteristics of the problem to be solved. The algorithm was set with the following hyperparameters: a population size of 50, up to 1000 generations, a recombination constant of 0.7 and a mutation constant dithering from 0.5 to 1.

### Training of a decision set classifier

All rules mined in the previous step were also applied to neutral gene pairs (N) to estimate the rule negative coverage ${\mathcal {C}}_{r}(N)$ for any rule *r*.

Using these rules, we trained a decision set (DS) model [47], a type of associative classifier [50] based on a collection of unordered rules interpreted as disjunction. Training a decision set consists of two phases: first, selecting a representative subset of rules out of an initial rule set and second, estimating the class probabilities associated with each model decision.

We implemented the first phase with the weighted set cover algorithm, inspired by the RUDIK rule mining method [38]. This greedy heuristic can find a representative subset of rules in reasonable time constraint. In this approach, a weight is assigned to a candidate rule set (Eq. (4)), with lower weights given to rule sets that have a high coverage of disease-causing gene pairs (${\mathcal {C}}_{R}(D)$) and a low coverage of neutral gene pairs (${\mathcal {C}}_{R}(N)$). We adapted the coverage calculation to take into account instance weights. A parameter $\alpha \in [0,1]$ calibrates the relative importance of the positive coverage or negative coverage.

4 $$\begin{aligned} w(R)=\alpha \cdot \left( 1-\frac{\left| {\mathcal {C}}_{R}(D)\right| }{|D|}\right) + (1-\alpha ) \cdot \left( \frac{\left| {\mathcal {C}}_{R}(N)\right| }{\left| N\right| }\right) \end{aligned}$$

This algorithm also defines a marginal weight $w_{m}$ (Eq. (5)), that quantifies the weight increase by adding a rule *r* to the decision set of rules *R*.

5 $$\begin{aligned} w_{m}(r)=w\left( R \cup \{r\}\right) -w(R) \end{aligned}$$

The greedy procedure starts with an empty decision set solution *R*. Then, at each iteration, it picks the rule from the original rule set with the minimum marginal weight and adds it to the solution *R*. The procedure stops when the marginal weight is greater than or equal to 0.

The selected rules were then used to build a predictive model. We assigned to each rule a probability estimate for the disease-causing class label ($l_D$), defined in Equation (6). This estimate corresponds to the precision of the rule corrected by the class imbalance in the training dataset.

6 $$\begin{aligned} p(l_D|r)=\frac{\left| {\mathcal {C}}_{r}(D)\right| }{\left| {\mathcal {C}}_{r}(D)\right| + \frac{\left| D\right| }{\left| N\right| } \cdot \left| {\mathcal {C}}_{r}(N)\right| } \end{aligned}$$

The model decision process was set up according to these criteria: (1) if a gene pair matches multiple rules in the decision set, the rule with the highest probability estimate is chosen; (2) If a gene pair does not match any of the rules, it is predicted as neutral with a probability estimate based on uncovered training instances.

### Generation of knowledge-based explanations

Contextual explanations were generated by first obtaining the matching rules returned by the model for a predicted positive gene pair and then using these rules to query the KG. The returned paths were then transformed into a set of nodes and edges, forming a subgraph.

The subgraphs were subsequently saved in the Graph Markup Language (GraphML) format [100] and could be explored via the graph visualisation software Cytoscape [101].

### Supplementary Information

**Additional file 1**. Supplementary Tables and Figures (A-I).

## Data Availability

The BOCK knowledge graph generated and analysed during the current study is available in the Zenodo repository 10.5281/zenodo.8124854. We provide it as an RDF graph (Resource Description Framework) along with its data model as an OWL file. For additional compatibility with standard graph libraries and tools, we also provide BOCK in the Graph Markup Language (GraphML) as well as in tab separated files compatible for direct import into the Neo4J Graph Database. The ARBOCK framework is provided as an open-source software at https://github.com/oligogenic/ARBOCK. It includes all scripts necessary to make predictions and generate explanations, as well as scripts to train and evaluate new models. We provide a Python notebook to reproduce the presented plots and tables. The predictive models presented in this study and the predictive result files are available in the same repository, under the models/ folder.
