# Supplementary material for: A knowledge graph approach to predict and interpret disease-causing gene interactions
Source: BMC Bioinformatics. 2023 Aug 29;24:324. doi: 10.1186/s12859-023-05451-5 (PMC10463539; doi:10.1186/s12859-023-05451-5)
Supplement: Supplementary file 1 — Additional file 1. Supplementary Tables and Figures (A-I). [file 12859_2023_5451_MOESM1_ESM.pdf]

# Supplementary Information – A Knowledge Graph approach to predict and interpret Disease-causing Gene Interactions

Alexandre Renaux[1,2,3*], Chloé Terwagne[1,2], Michael Cochez[4,5], Ilaria Tiddi[4], Ann Nowé[1,3], Tom Lenaerts[1,2,3*]

[1*]Interuniversity Institute of Bioinformatics in Brussels, Université Libre de Bruxelles - Vrije Universiteit Brussel, Brussels, Belgium.
[2]Machine Learning Group, Université Libre de Bruxelles, Brussels, Belgium.
[3]Artificial Intelligence lab, Vrije Universiteit Brussel, Brussels, Belgium.
[4]Computer Science, Vrije Universiteit Amsterdam, Amsterdam, The Netherlands.
[5]Discovery Lab, Elsevier, Amsterdam, The Netherlands.

*Corresponding author(s). E-mail(s): Alexandre.Renaux@ulb.be; tom.lenaerts@ulb.be;
Contributing authors: chloe.terwagne@live.be; m.cochez@vu.nl; i.tiddi@vu.nl; ann.nowe@ai.vub.ac.be;

# Appendix A BOCK knowledge graph data integration

| Source database | Version | Extracted information | KG components |
|---|---|---|---|
| **OLIDA** [1] | 04-2022 | Oligogenic gene combinations, DOI, timestamp, ethnicity, curation scores | OLIDA |
| **Mentha** [2] | 10-10-2022 | Manually curated protein-protein interactions in human | PPI |
| **STRING db** [? ] | v11.5 | Human protein sequence raw blastp scores | SEQSIM |
| **TCSBN** [3] | 26-10-2020 | Tissue-specific co-expression network from normal human tissues (derived from GTEx) | COEXP |
| **GTEx** [4] | V8 | Median gene-level RNA-seq transcript per millions (TPM) by tissue ; Tissue names and sample sizes | COEXP |
| **InterPro** [4] | 87.0 | Human protein domains and families | DOMAIN, FAMILY |
| **CORUM** [? ] | 4.0 | Human protein complexes | COMPLEX |
| **Gene Ontology** [5] | 10-07-2022 | Gene ontology and annotations on human genes (positive associations with qualifiers "enables", "involved in", "is active in" and "located in"), excluding IEA and ND evidence codes | PROCESS, FUNCTION, CELLCMP |
| **Human Phenotype Ontology** [6] | 06-2022 | Human 'phenotypic abnormality' sub-ontology (non-obsolete), gene annotations and disease associations | PHENO, DISEASE |
| **dbNSFP** [7] | v4.3 | RVIS [8] and GDI [9] gene scores | All with Gene |
| **HGNC** [10] | 2022-10-12 | Official gene names and Ensembl mappings | All with Gene |
| **Ensembl** [11] | Release 107 | Ensembl identifiers, gene name and entrez id mappings | All with Gene |
| **UniProt** [12] | 2022_04 | UniProt mappings to Ensembl identifiers | PPI, SEQSIM, COMPLEX, DOMAIN, FAMILY |

**Table A1. Source databases to construct BOCK**. Bioinformatics databases used with their version used for this study. The specific extracted information from each database is indicated as well as the corresponding KG component it is used for (see Supplementary Table A2).

| KG component | Node types | Edge types | Edge score | Filtering |
|---|---|---|---|---|
| **OLIDA** | OligogenicCombination, Disease | causes | None | None |
| | OligogenicCombination, Gene | involves | None | None |
| **DISEASE** | Disease, Phenotype | described | Disease-Phenotype frequency | None |
| **PPI** | Gene | physInteracts | Original Mentha score | None |
| **SEQSIM** | Gene | seqSimilar | Blast Score Ratio (BSR) | align. coverage $\geq$ 50% ; BSR $\geq$ 0.2 |
| **COEXP** | Gene | coexpresses | $CI_{low}$(cor) | tissue-sample-size > 70 ; $Z_{tpm}(G_1)$ & $Z_{tpm}(G_2) \geq$ -3 ; $CI_{low}(cor) \geq 0.80$ ; p-val-adj < 0.01 |
| **DOMAIN** | ProteinDomain, Gene | hasUnit | $FI(PD)$ | Only "*Active_site*", "*Binding_site*", "*Conserved_site*", "Domain", "PTM", "Repeat" |
| **FAMILY** | ProteinFamily, Gene | belongs | $FI(PF)$ | Only "Family" and "*Homologous_superfamily*" |
| **COMPLEX** | ProteinComplex, Gene | forms | $FI(PC)$ | None |
| **PROCESS** | BiologicalProcess, Gene | associated | $FI(BP)$ | None |
| | BiologicalProcess | resembles | $SimGIC(BP_1, BP_2)$ | Sim. score $\geq$ 0.5 |
| **FUNCTION** | MolecularFunction, Gene | associated | $FI(MF)$ | None |
| | MolecularFunction | resembles | $SimGIC(MF_1, MF_2)$ | Sim. score $\geq$ 0.5 |
| **CELLCMP** | CellularComponent, Gene | associated | $FI(CC)$ | None |
| | CellularComponent | resembles | $SimGIC(CC_1, CC_2)$ | Sim. score $\geq$ 0.5 |
| **PHENO** | Phenotype, Gene | associated | $FI(P)$ | None |
| | Phenotype | resembles | $SimGIC(P_1, P_2)$ | Sim. score $\geq$ 0.5 |

**Table A2. KG components integration into BOCK.** Data integration of the BOCK knowledge graph by component. Each component describes a different biological level or view and has been integrated into the knowledge graph from a source network or ontology database (see Supplementary Table A1) into KG node types and edge types. For most components, an edge score is computed and used as an edge property. For some components, the integration process has required the application of a filtering stage to limit the noise and the size of the integrated network. More details about the computation of scores and filtering steps are provided in the Methods section BOCK data integration and resources.

| Network resource | # Genes | # Exclusive genes |
|---|---|---|
| Domains (InterPro) | 20302 | 691 |
| Functions (GO) | 18344 | 46 |
| PPI (Mentha) | 17062 | 89 |
| Coexpression (TSCBN) | 14940 | 33 |
| Seq. similarity (STRING) | 12226 | 67 |
| Phenotypes (HPO) | 4870 | 32 |
| Complexes (CORUM) | 4357 | 0 |
| Oligogenic combinations (OLIDA) | 907 | 0 |

**Table A3. Gene contribution from BOCK sources**. BOCK source network gene contributions. We calculated #*Genes* as the total number of genes present in each source network. #*Exclusive genes* was obtained by considering genes that are exclusively present in each of the specific network resource.

# Appendix B Disease-causing gene pair details

This appendix provides detailed information on the selection and analysis of disease-causing gene pairs. It covers the assignment of oligogenic evidence levels to each variant combination (Supplementary Table B1), the selection process of pathogenic gene pairs based on these levels (Supplementary Table B2), and specifics of the independent test set of 15 selected pathogenic gene pairs (Supplementary Table B3).

| Evidence level | Weight | Criteria based on OLIDA curation scores | | | | |
|---|---|---|---|---|---|---|
| | | FAMmanual | OR | (STATmeta | AND | STATmanual) |
| **Weak** | 0.33 | 1 | \|\| | 1 | & | 1 |
| **Moderate** | 0.67 | 2 | \|\| | 2 | - | - |
| **Strong** | 1.00 | 3 | \|\| | 3 | - | - |

**Table B1. Oligogenic evidence levels based on familial and statistical scores.** For each variant combination present in OLIDA, different types of curation confidence scores have been assigned according to the strength of evidences provided (*e.g.* relatives genotype information, cohort statistical analyses, etc). See [1] for more information on OLIDA confidence scores. For this method, we created an *Evidence level*, solely based on familial (FAMmanual) and statistical (STATmeta, STATmanual) curation confidence scores, associated with a linear weight. If none of the criteria are matching, the variant combination is discarded.

| Status | # variant combinations | # gene pairs | # diseases |
|---|---|---|---|
| **Discarded** | 324 | 130 | 71 |
| **Weak** | 577 | 280 | 108 |
| **Moderate** | 131 | 101 | 71 |
| **Strong** | 86 | 60 | 30 |
| **Selected** | 794 | **441** | 153 |

**Table B2. Pathogenic gene pair selection according to evidence levels**. Evidence levels have been attributed for each digenic variant combination in OLIDA (Supplementary Table B1). All variant combinations passing at least the *Weak* evidence level criteria have been selected (a total of 794) and weighted accordingly and the remaining variant combinations have been discarded. Statistics are presented with a gene-level and disease-level aggregation, by attributing the highest evidence level of each set.

| Gene pair | Evidence level | Disease | PMID | Publication date |
|---|---|---|---|---|
| MYH7-ANKRD1 | Moderate | Left ventricular noncompaction | 34752814 | 06/11/2021 |
| LAMA3-LAMB3 | Moderate | Severe generalized junctional epidermolysis bullosa | 34837689 | 01/10/2021 |
| TSHR-SLC26A4 | Moderate | Congenital hypothyroidism | 34374102 | 10/08/2021 |
| JAG1-DUOXA1 | Moderate | Congenital hypothyroidism | 34374102 | 10/08/2021 |
| CDCA8-DUOX2 | Moderate | Congenital hypothyroidism | 34374102 | 10/08/2021 |
| HOXB3-TG | Moderate | Congenital hypothyroidism | 34374102 | 10/08/2021 |
| MYO7A-SHROOM2 | Strong | Atypical hemolytic uremic syndrome | 34391192 | 02/08/2021 |
| PKHD1-PKD1 | Moderate | Autosomal recessive polycystic kidney disease | 34032358 | 25/05/2021 |
| POLG-PPFIA4 | Moderate | Isolated focal cortical dysplasia | 34095804 | 07/05/2021 |
| SLC20A2-PDGFRB | Moderate | Bilateral striopallidodentate calcinosis | 33793087 | 01/04/2021 |
| TRAPPC11-TTN | Strong | Limb-girdle muscular dystrophy | 33746696 | 04/03/2021 |
| SPG7-SPAST | Strong | Hereditary spastic paraplegia | 33598982 | 20/02/2021 |
| MITF-C2orf74 | Moderate | Waardenburg syndrome | 33571247 | 12/02/2021 |
| CHD7-CDON | Moderate | Kallmann syndrome | 33208564 | 17/11/2020 |
| BMPR2-NOTCH3 | Moderate | Idiopathic pulmonary arterial hypertension | 33007923 | 30/09/2020 |

**Table B3. Independent test set details**. Details on the selected held-out test set made of 15 pathogenic gene pairs. This test set was selected based on an automatic procedure designed to favour diverse, confident and recently published cases: first, all disease-causing gene pairs were ranked by their first associated article publication date, then for each gene pair from the most recent to the oldest, gene pairs were chosen if their evidence level was at least Moderate (Supplementary Table B1) and if none of their genes overlapped with the previously selected ones.

# Appendix C ARBOCK framework parameter analysis

We present here in more details the parameters used in the ARBOCK framework (Table C1) and how their choice was empirically determined. In particular, we analyse the effect of the `alpha`, `minsup_ratio` and `max_rule_length` parameters on the model's performance, measured by the Area Under the Receiver Operating Characteristic (AUROC), and explanation complexity, measured mainly by the number of paths on average present in the explanation subgraph, for True Positive (TP) instances. Note that for this benchmark, the choice of AUROC as performance metric stems from its ability to meaningfully evaluate and compare models irrespective of class imbalance and threshold settings, without being artificially inflated by models that merely predict the majority class (*e.g* sets of parameters leading to a model with only the default rule), considering our high class imbalance setting.

Figure C2 present a comprehensive analysis of the best performing models (considering all other parameters) according to the alpha parameter of the decision set model. At $\alpha$=0.5, performance is maximised and higher values lead to more explanation complexity, measured as the number of matching rules and explanation path count. This model corresponds to `path_cutoff`=3 ; `minsup_ratio`=0.2 ; `max_rule_length`=3.

Figure C3 shows the combined effect of `minsup_ratio` and `max_rule_length`, for fixed $\alpha$=0.5 and `path_cutoff`=3, on performance and explanation path count. Higher `max_rule_length` leads to a similar performance but at the cost of increasing explanation complexity. Lower `minsup_ratio` leads to better performance and a smaller number of paths in explanations.

Fixing `path_cutoff`=2, as presented in Figures C4 and C5 leads to a maximum performance of 0.67 for all evaluated sets of parameters but yields simpler explanations in terms of path count.

| **Parameter** | **Description** | **Step** | **Algorithm** | **Range** | **Set value** |
|---|---|---|---|---|---|
| `path_cutoff` | Maximum number of edges traversed for a path | Path extraction | Depth-first search | [1, $\infty$[ | 3 |
| `minsup_ratio` | Minimum fraction of entity pairs to consider a pattern frequent | Rule mining | Weighted apriori | ]0, 1[ | 0.2 |
| `max_rule_length` | Maximum number of metapaths in a rule | Rule mining | Weighted apriori | [2, $\infty$[ | 3 |
| $\alpha$ | Relative importance of positive coverage over negative coverage | Model training | Greedy weighted set cover | ]0, 1[ | 0.5 |

**Table C1. Framework parameters summary**. This table presents the parameters defined in the ARBOCK approach. For each parameter, a description, its relevant step in the process, the algorithm employed in that step, the range of potential values, and the specific value chosen for this study are provided.

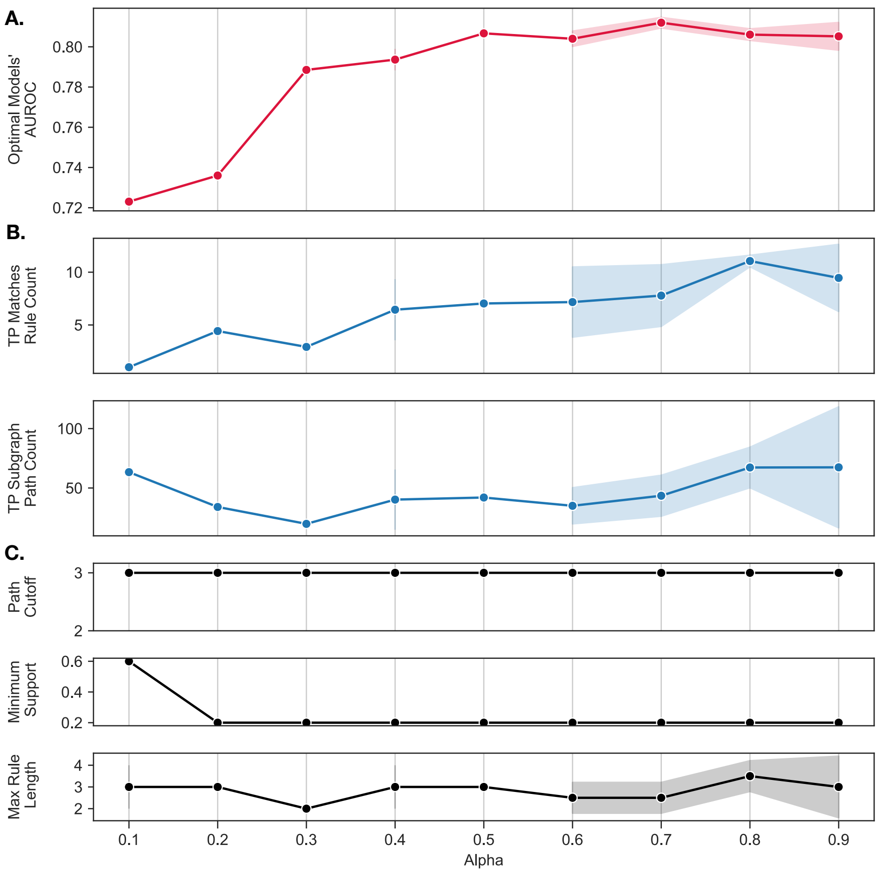


**Fig. C2. Influence of decision model's alpha parameter tuning** Evaluating decision set models over a spectrum of the decision model's alpha ($\alpha$) values (0.1-0.9) and variable parameters - Path Cutoff (`path_cutoff`) {2,3}, Minimum Support (`minsup_ratio`) {0.2, 0.4, 0.6, 0.8}, Max Rule Length (`max_rule_length`) {2,3,4}, we highlight the performance and characteristics of the optimal models for each alpha value. This evaluation is performed on the model excluding Phenotype, prone to study bias. Optimal models for each alpha are selected based on mean AUROC from a stratified cross-validation, keeping all within 0.01 of the highest performer to ensure comparable accuracy levels. **(A)** Mean AUROC of top-performing models evaluated via stratified cross-validation on test sets **(B)** Measures of explanation complexity for the associated models, calculated from predicted true positive (TP) gene pairs on the test sets: Rule Count measures the mean number of rules per match, and Path Count measures the mean number of paths across all rules, on average per match. **(C)** Parameter distribution for the corresponding top-performing models.

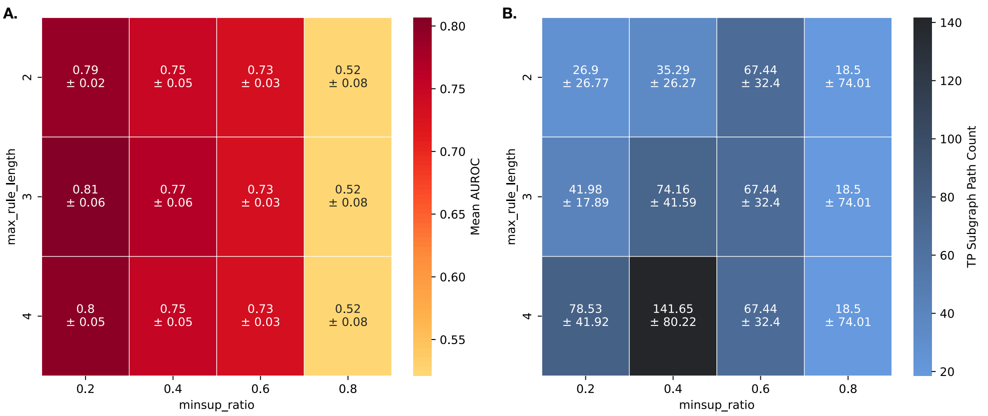


**Fig. C3. Influence of minimum support and max rule length parameters tuning.** Heatmaps showing the influence of parameters `minsup_ratio` and `max_rule_length` on the performance and explanation complexity for decision models trained with paths excluding Phenotype and with fixed parameters: $\alpha = 0.5$ and `path_cutoff` $= 3$.
**(A)** Effect on the model performance, evaluated with mean AUROC on a stratified cross-validation. **(B)** Effect on the model explanation complexity, evaluated with the Path Count measure, measuring the mean number of paths across all rules, on average per True Positive matches.

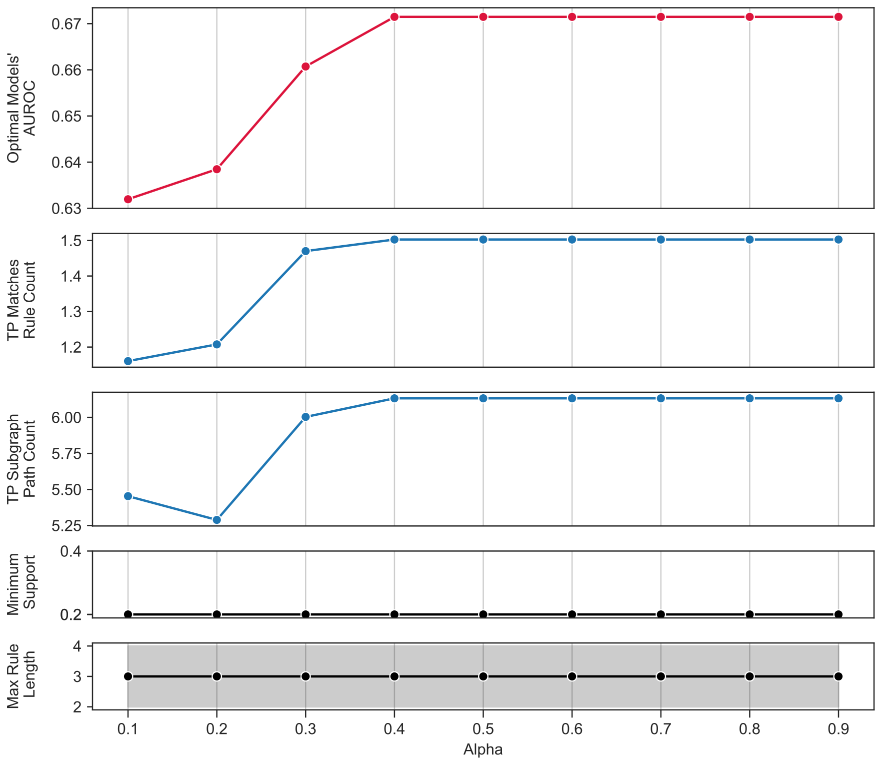


**Fig. C4. Influence of decision model's alpha parameter tuning with path_cutoff=2** Evaluating decision set models with a fixed Path Cutoff = 2, over a spectrum of the decision model's alpha ($\alpha$) values (0.1-0.9) and variable parameters - Minimum Support (`minsup_ratio`) {0.2, 0.4, 0.6, 0.8}, Max Rule Length (`max_rule_length`) {2,3,4}, we highlight the performance and characteristics of the optimal models for each alpha value. This evaluation is performed on the model excluding Phenotype, prone to study bias. Optimal models for each alpha are selected based on mean AUROC from a stratified cross-validation, keeping all within 0.01 of the highest performer to ensure comparable accuracy levels. **(A)** Mean AUROC of top-performing models evaluated via stratified cross-validation on test sets **(B)** Measures of explanation complexity for the associated models, calculated from predicted true positive (TP) gene pairs on the test sets: Rule Count measures the mean number of rules per match, and Path Count measures the mean number of paths across all rules, on average per match. **(C)** Parameter distribution for the corresponding top-performing models.

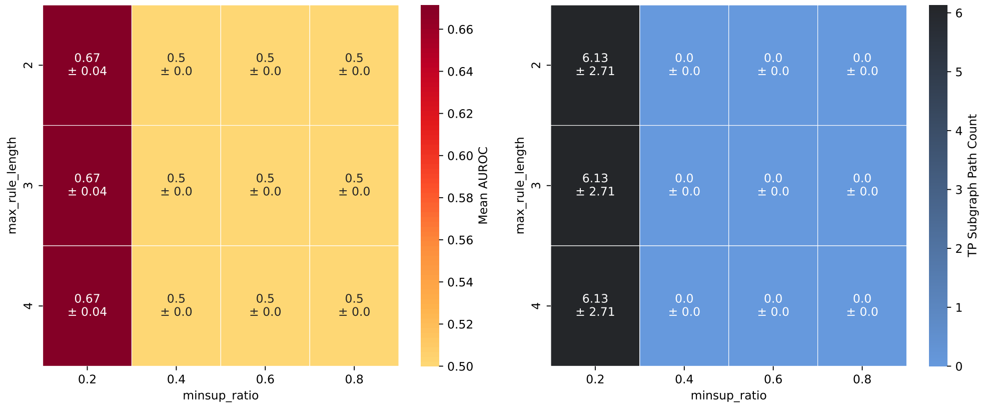


**Fig. C5. Influence of minimum support and max rule length parameters tuning, with path_cutoff = 2**. Heatmaps showing the influence of parameters `minsup_ratio` and `max_rule_length` on the performance and explanation complexity for decision models trained with paths excluding Phenotype and with fixed parameters: $\alpha = 0.5$ and `path_cutoff` $= 2$. **(A)** Effect on the model performance, evaluated with mean AUROC on a stratified cross-validation. **(B)** Effect on the model explanation complexity, evaluated with the Path Count measure, measuring the mean number of paths across all rules, on average per True Positive matches.

# Appendix D Mined rules metrics distribution

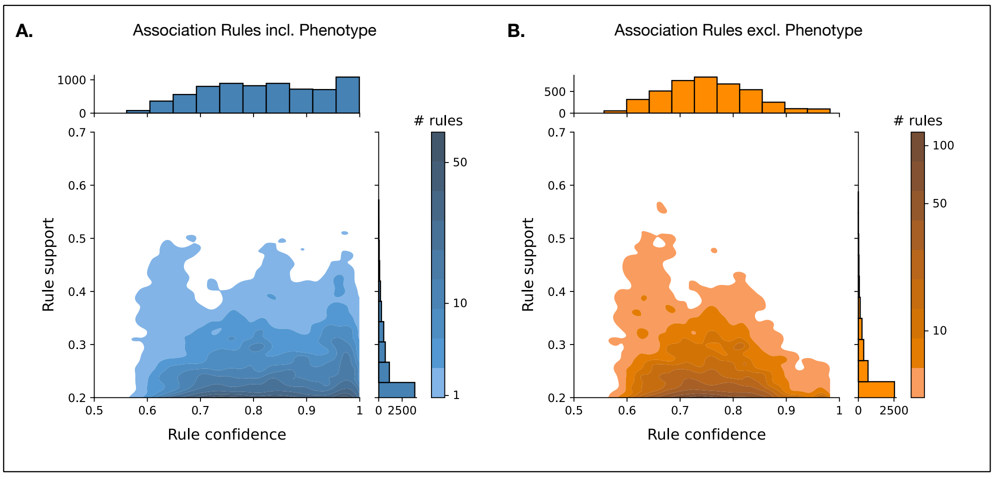


**Fig. D1.** Distribution of all candidate association rules obtained by mining all disease-causing gene pairs in the training set, according to two metrics: the rule support (*i.e.* the ratio of pathogenic gene pairs associated with the rule pattern) and the rule confidence (*i.e.* the likelihood of a gene pair being pathogenic when it matches the rule pattern). The darkness of the shade increases logarithmically with the number of rules. **A.** Rules mined from paths including Phenotype (*incl. Pheno*). **B.** Rules mined from paths excluding Phenotype (*excl. Pheno*).

# Appendix E Overview of the machine-learning model assessment

| DOME | Version | 1.0 |
|---|---|---|
| **Data** | Provenance | OLIDA [1], Mentha [2], STRING db [**?** ], TCSBN [3], GTEx [4], InterPro [4], CORUM [**?** ], Gene Ontology [5], Human Phenotype Ontology [6], dbNSFP [7], HGNC [10], Ensembl [11], UniProt [12]. All these sources are merged as the knowledge graph BOCK. Negative gene pair data are generated from the 1000 genomes Project [**?** ]. |
| | Dataset splits | 426 positive instances, 42.600 negative instances for training data. 15 positive instances as validation set. |
| | Redundancy between data splits | No overlap |
| | Availability of data | Yes: BOCK knowledge graph at: doi.org/10.5281/zenodo.7185679 and 1000 genome project at: www.internationalgenome.org |
| **Optimization** | Algorithm | Associative classification with a weighted set cover approach. |
| | Meta-predictions | No. |
| | Data encoding | Transformation of gene pair paths into metapath-based rules. |
| | Parameters | path cutoff = 3 ; minsup ratio = 0.2 ; max rule length = 3 ; $\alpha = 0.5$ |
| | Features | Metapath features obtained by aggregating the path information for all gene pairs of the positive set. |
| | Fitting | To avoid overfitting, rules can only associate 3 different metapaths and needs to be supported by at least 20% of positive training instances. |
| | Regularization | No |
| | Availability of configuration | Yes: github.com/oligogenic/bock_rule_mining |
| **Model** | Interpretability | White box model (rule-based) with knowledge-based explanations for gene pairs predicted as positive. |
| | Output | Classification probability (with predicted class) and explanation subgraphs for positively predicted instances |
| | Execution time | 1000 samples in 0.56 seconds on a single Intel i5 core. |
| | Availability of software | Yes, Github: github.com/oligogenic/bock_rule_mining |
| **Evaluation** | Evaluation method | Both stratified 10-fold cross validation and evaluation on 15 independent validation positive instances |
| | Performance measures | Precision, Recall, ROC AUC, PR AUC |
| | Comparison | Model with and without phenotype information and the DiGePred model on the independent validation set. |
| | Confidence | Stability of the ROC AUC measure over different folds of cross-validation (std. 0.03). |
| | Availability of evaluation | Yes: Github: github.com/oligogenic/bock_rule_mining |

**Table E1.** DOME recommendation table consisting of essential information to assess the machine learning approach [13]. These criteria correspond to the decision set models presented in this study.

# Appendix F Predictive performance of a Random Walk with Restart on BOCK

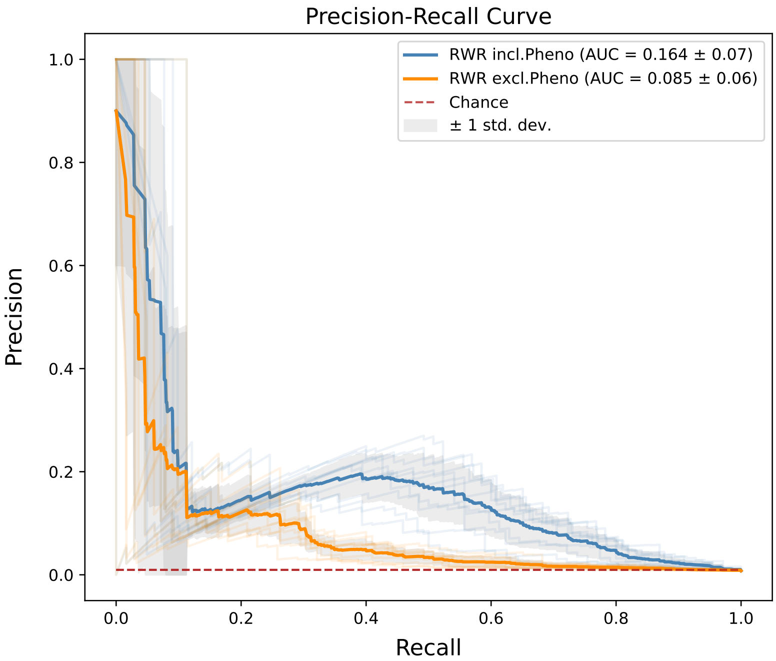


**Fig. F1.** For baseline comparison, a random walk with restart (with restart probability = 0.7 [14]) was performed on BOCK for all selected gene pairs starting from the gene with the lowest Residual Variant Intolerance Score (RVIS) [8] to the gene with the highest. A logistic regression model trained with the random walk diffusion probability as feature was trained. We evaluated the model performance on predicting gene pair pathogenicity on a 10-fold stratified cross-validation and we report the Precision Recall Curve for random walks including and excluding the traversal of Phenotype nodes.

# Appendix G Knowledge biases and the influence of phenotype inclusion

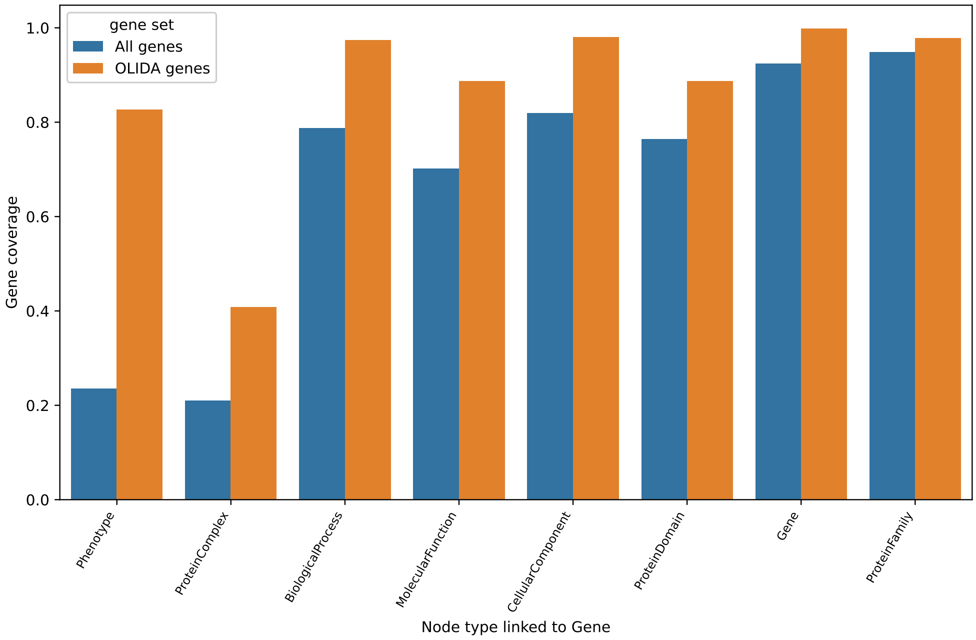


**Fig. G1. Gene annotation coverage in OLIDA vs. all genes**. Ratio of genes linked to each node types in the knowledge graph (excluding OligogenicCombination and Disease). In blue, this ratio is calculated over all human genes. In orange, it is calculated over all genes involved in a combination in OLIDA. The node types are ordered by decreasing difference between these two ratios.

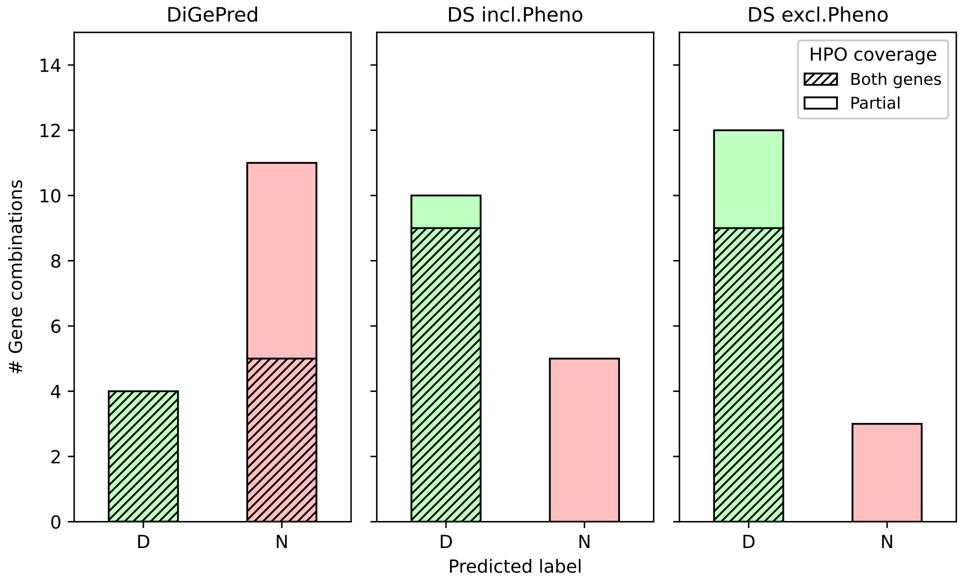


**Fig. G2. Comparison of predictions on held-out pathogenic gene pairs with different phenotype annotations.** Comparison of three predictive models: "DiGePred" [15] (a gene pair pathogenicity predictor with features at the gene and relationship level, including strong phenotype-based features), "BOCK-DS" (the decision set trained without phenotype-based paths) and "BOCK-DS incl. Phenotype" (the decision set trained with inclusion of paths traversing nodes of type "Phenotype"). The 15 held-out disease-causing gene combinations are evaluated on the three models and the number of gene pairs is shown for each predicted label (D=Disease-causing ; N=Neutral) ; The overlaid stripes indicates that both genes of a combination are annotated with at least one human phenotype ontology (HPO) term.

| Gene pair (A,B) | #Pheno. A | #Pheno. B | Phenotype Similarity | DiGePred | DS incl.Pheno | DS excl.Pheno |
|---|---|---|---|---|---|---|
| HOXB3-TG | 0 | 32 | 0.00 | 0.00 | 0.06 | 0.80 |
| *CDCA8-DUOX2* | 0 | 38 | 0.00 | 0.01 | 0.92 | 0.84 |
| *MYO7A-SHROOM2* | 34 | 0 | 0.00 | 0.02 | 0.98 | 0.97 |
| *MITF-C2orf74* | 58 | 0 | 0.00 | 0.02 | 0.06 | 0.12 |
| *JAG1-DUOXA1* | 96 | 0 | 0.00 | 0.01 | 0.06 | 0.12 |
| *POLG-PPFIA4* | 245 | 0 | 0.00 | 0.03 | 0.06 | 0.12 |
| *BMPR2-NOTCH3* | 19 | 133 | 0.01 | 0.05 | 0.98 | 0.93 |
| *MYH7-ANKRD1* | 171 | 8 | 0.05 | 0.23 | 1.00 | 0.97 |
| *PKHD1-PKD1* | 75 | 27 | 0.10 | 0.03 | 1.00 | 0.97 |
| *CHD7-CDON* | 217 | 116 | 0.10 | 0.38 | 1.00 | 0.84 |
| *TRAPPC11-TTN* | 73 | 114 | 0.11 | 0.26 | 0.99 | 0.89 |
| *SPG7-SPAST* | 53 | 34 | 0.21 | 0.66 | 1.00 | 0.80 |
| *TSHR-SLC26A4* | 60 | 35 | 0.28 | 0.88 | 1.00 | 0.80 |
| *SLC20A2-PDGFRB* | 35 | 108 | 0.32 | 0.72 | 1.00 | 0.80 |
| *LAMA3-LAMB3* | 71 | 74 | 0.84 | 0.90 | 1.00 | 0.97 |

**Table G3. Independent test set predicted probabilities in relation with phenotype**. Table presenting the 15 pathogenic gene pairs held-out as independent test set. For each gene pair, the number of phenotype terms (HPO) linked to to the first and second gene is shown, as well as the Jaccard similarity index between HPO terms of the two genes (Phenotype Similarity). The predicted probabilities are shown for the three benchmarked models: "DiGePred" [15] (a gene pair pathogenicity predictor with features at the gene and relationship level, including strong phenotype-based features), "DS incl.Pheno" (the decision set trained with inclusion of paths traversing nodes of type "Phenotype") and "DS excl.Pheno" (the decision set trained without phenotype-based paths). Green and red colors represent the predicted classification labels, respectively "disease-causing" and "neutral", based on the classification optimal thresholds of these models (DiGePred=0.496 ; DS incl.Pheno=0.929 ; DS excl.Pheno=0.788). Rows are ordered by the Phenotype similarity. The influence of phenotype annotations over gene pairs can be observed for the "DiGePred" and the "DS incl.Phenotype" models, both reliant on phenotype-related features.

# Appendix H Metaedge ablation study

The biological knowledge graph, BOCK, contains a multitude of biological relationships represented as different types of edges, also referred to as metaedges (as summarised in Table 1). In this experiment, we seek to understand the contribution of each metaedge in both the predictive performance and the complexity of the explanations provided by decision set models, by comparing models trained with different versions of BOCK omitting one metaedge.

We first assessed the baseline performance and complexity on a 10-fold stratified cross-validation, similarly as done when evaluating *DS excl. Pheno*, described in Results (excluding Phenotype-based metaedges, *i.e* $GaP$ and $PrP$ metaedges). Using this baseline setting as reference, we evaluate, in a similar manner, decision models based on BOCK excluding an additional metaedge, from the list of remaining metaedges taken into consideration. For each metaedge-excluded model, we report metrics of predictive performance (Figure H1) and explanation complexity (Figure H2), based on the 10 test folds.

Note that explanation complexity here refers to the average number of paths obtained for explanations of True Positive gene pairs. A lower number of path leads to explanations that are easier to interpret.

From this experiment, we can observe that eliminating physical interaction edges ($GpG$) and associations with biological processes ($GaBP$) from BOCK adversely affects classifier performance. Removing other metaedges has a negligible effect on predictive performance. Interestingly, omitting gene functional relationships results in a higher average number of paths yielded in explanations, possibly due to an increased prevalence of gene-gene relationships in rules. The removal of the coexpression relationship $GeG$ reduces the number of paths in the provided explanations, likely owing to the high frequency of $GeG$ edges in BOCK.

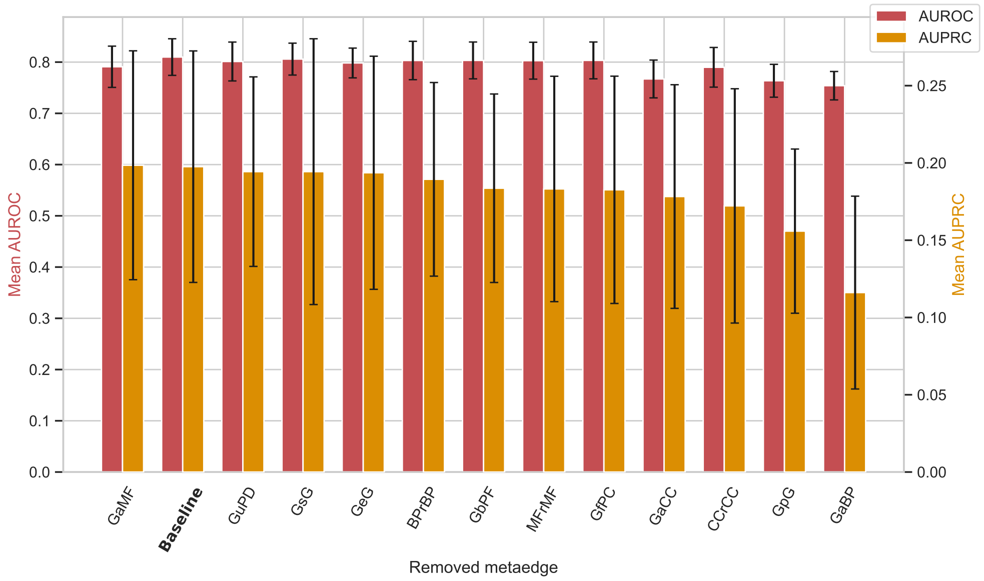


**Fig. H1. Influence of BOCK metaedge ablation on performance**. Considering a decision set model, its predictive performance is estimated by the Mean Area Under the Receiver Operating Characteristic Curve (AUROC, red) and the Mean Area Under the Precision-Recall Curve (AUPRC, orange). These metrics are evaluated on a 10-fold stratified cross-validation. The 'Baseline' bar (bold) represents the distribution of this metric for a model excluding Phenotype relationship ($GaP$ metaedge). Based on that baseline, all other bars represent models where an additional metaedge (type of edge, as summarised in 1) has been removed from BOCK. Error bars indicate the standard deviation of this metric for all evaluated test folds.

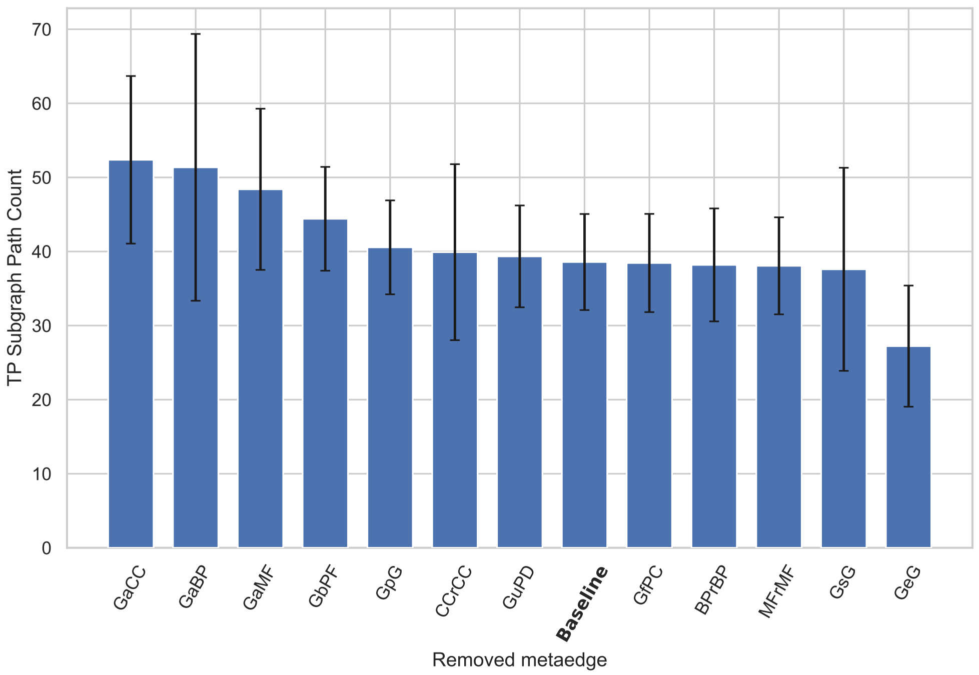


**Fig. H2. Influence of BOCK metaedge ablation on explanation complexity**. Considering a decision set model, the explanation complexity is estimated by the mean count of paths of explanation subgraphs returned for the True Positive (TP) gene pairs. This metric is evaluated on a 10-fold stratified cross-validation. The 'Baseline' bar (bold) represents the distribution of this metric for a model excluding Phenotype relationship ($GaP$ metaedge). Based on that baseline, all other bars represent models where an additional metaedge (type of edge, as summarised in 1) has been removed from BOCK. Error bars indicate the standard deviation of this metric for all evaluated test folds.

# Appendix I Additional predictive explanations

The three figures below showcase the remaining graphical explanations for the $3^{rd}$, $4^{th}$ and $5^{th}$ rules (in order of predicted probability), matching the digenic gene pair *MYH7-ANKRD1* in decreasing order of predicted probability (see Figure 6).

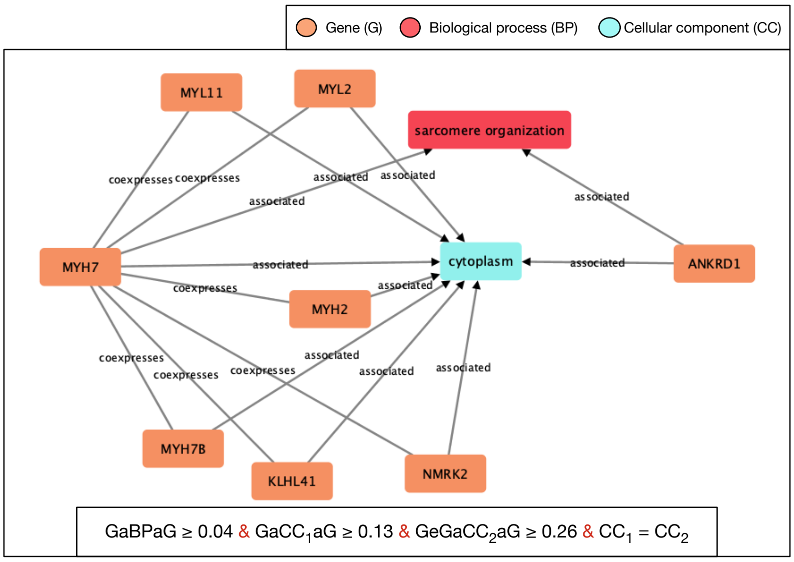


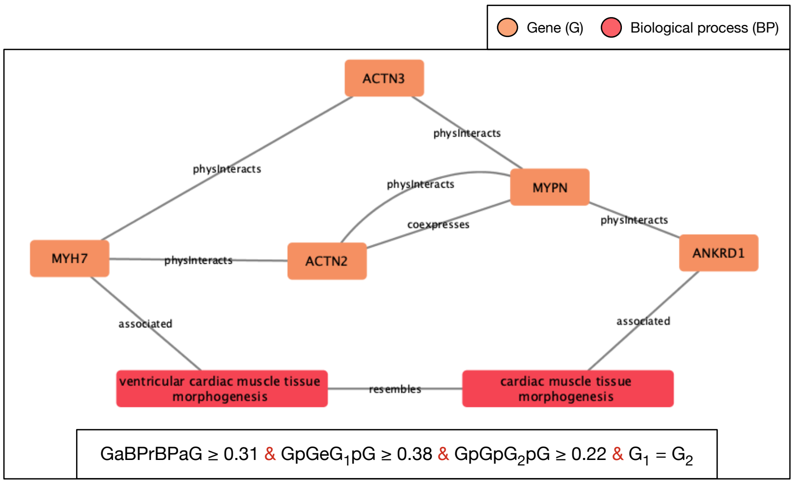

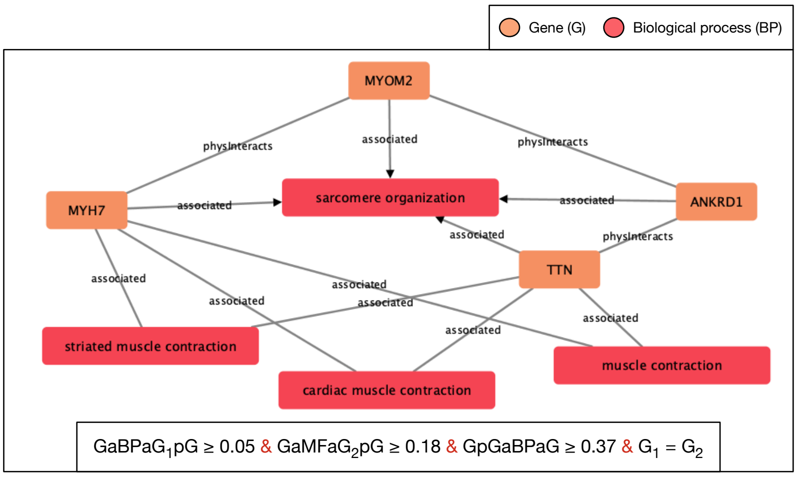


Each rule is written in their abbreviated form (see Table 1) with its conditions separated by &. Indices for node types (*e.g.* $G_1$) are used in unification conditions (*e.g.* $G_1$=$G_2$) to constrain entities to be the same across different metapaths. The numerical value associated to each metapath (*e.g.* $\geq 0.31$) sets the path reliability threshold, which conditions the minimum path reliability score of all underlying paths.

# References

[1] Nachtegael C, Gravel B, Dillen A, Smits G, Nowé A, Papadimitriou S, et al. Scaling up oligogenic diseases research with OLIDA: The Oligogenic Diseases Database. Database. 2022;2022(December 2021):1–15. https://doi.org/10.1093/database/baac023.

[2] Calderone A, Castagnoli L, Cesareni G.: Mentha: A resource for browsing integrated protein-interaction networks.

[3] Lee S, Zhang C, Arif M, Liu Z, Benfeitas R, Bidkhori G, et al. TCSBN: A database of tissue and cancer specific biological networks. Nucleic Acids Research. 2018;46(D1):D595–D600. https://doi.org/10.1093/nar/gkx994.

[4] Aguet F, Barbeira AN, Bonazzola R, Brown A, Castel SE, Jo B, et al. The GTEx Consortium atlas of genetic regulatory effects across human tissues. Science. 2020;369(6509):1318–1330. https://doi.org/10.1126/SCIENCE.AAZ1776.

[5] The Gene Ontology C, That I, Acencio M, Lægreid A, Kuiper M, Among O. The Gene Ontology Resource: 20 years and still GOing strong. Nucleic Acids Research. 2019;8(47):D330—-D338. https://doi.org/10.17863/CAM.36439.

[6] Köhler S, Gargano M, Matentzoglu N, Carmody LC, Lewis-Smith D, Vasilevsky NA, et al. The human phenotype ontology in 2021. Nucleic Acids Research. 2021;49(D1):D1207–D1217. https://doi.org/10.1093/nar/gkaa1043.

[7] Liu X, Li C, Mou C, Dong Y, Tu Y. dbNSFP v4: a comprehensive database of transcript-specific functional predictions and annotations for human nonsynonymous and splice-site SNVs. Genome Medicine. 2020 Dec;12(1):1–8. https://doi.org/10.1186/s13073-020-00803-9.

[8] Petrovski S, Wang Q, Heinzen EL, Allen AS, Goldstein DB. Genic Intolerance to Functional Variation and the Interpretation of Personal Genomes. PLoS Genetics. 2013 Aug;9(8):e1003709. https://doi.org/10.1371/journal.pgen.1003709.

[9] Itan Y, Shang L, Boisson B, Patin E, Bolze A, Moncada-Vélez M, et al. The human gene damage index as a gene-level approach to prioritizing exome variants. Proceedings of the National Academy of Sciences of the United States of America. 2015 Nov;112(44):13615–13620. https://doi.org/10.1073/pnas.1518646112.

[10] Tweedie S, Braschi B, Gray K, Jones TEM, Seal RL, Yates B, et al. Genenames.org: The HGNC and VGNC resources in 2021. Nucleic Acids Research. 2021;49(D1):D939–D946. https://doi.org/10.1093/nar/gkaa980.

[11] Howe KL, Achuthan P, Allen J, Allen J, Alvarez-Jarreta J, Ridwan Amode M, et al. Ensembl 2021. Nucleic Acids Research. 2021 Jan;49(D1):D884–D891. https://doi.org/10.1093/nar/gkaa942.

[12] Bateman A. UniProt: A worldwide hub of protein knowledge. Nucleic Acids Research. 2019;47(D1):D506–D515. https://doi.org/10.1093/nar/gky1049.

[13] Walsh I, Fishman D, Garcia-Gasulla D, Titma T, Pollastri G, Capriotti E, et al. DOME: recommendations for supervised machine learning validation in biology. Nature Methods. 2021;18(10):1122–1127. https://doi.org/10.1038/s41592-021-01205-4.

[14] Valdeolivas A, Tichit L, Navarro C, Perrin S, Odelin G, Levy N, et al. Random walk with restart on multiplex and heterogeneous biological networks. Bioinformatics. 2019 Feb;35(3):497–505. https://doi.org/10.1093/bioinformatics/

bty637.
[15] Mukherjee S, Cogan JD, Newman JH, Phillips JA, Hamid R, Meiler J, et al. Identifying digenic disease genes via machine learning in the Undiagnosed Diseases Network. American Journal of Human Genetics. 2021 Jan;108(10):1946–1963. https://doi.org/10.1016/j.ajhg.2021.08.010.
